# Degradation of Hydrophobic Recycled Fine Aggregate Concrete Under Chloride Salt Dry–Wet Cycling Environment

**DOI:** 10.3390/ma19163469

**Published:** 2026-08-17

**Authors:** Yuwei Lu, Chunhong Chen, Xiaolin Zhang, Jianlei Liang, Xiang Guo

**Affiliations:** School of Urban Construction, Changzhou University, Changzhou 213164, China; 19895436350@163.com (Y.L.); zhang0708930@163.com (X.Z.); 17715339310@163.com (J.L.); guoxiang2131@cczu.edu.cn (X.G.)

**Keywords:** sodium methyl silicate, chloride dry–wet cycling, free chloride content, recycled fine aggregate concrete, silane-based network film, hydrophobicity

## Abstract

Reinforced concrete structures in marine environments are subjected to severe deterioration, particularly in tidal zones. The development of intrinsically hydrophobic concrete through internal modification provides a promising strategy to mitigate this challenge. This study employed sodium methyl silicate (SMS) as a hydrophobic agent to prepare recycled fine aggregate concrete (RFAC), which was subsequently subjected to accelerated indoor chloride dry–wet cycling. The deterioration behavior of RFAC and the degradation mechanism of the SMS-induced hydrophobic film during dry–wet cycling were investigated through evaluations of mechanical performance, hydrophobicity, chloride resistance, microstructure, phase composition, pore structure, chemical bonding, and functional groups. The results show that SMS improves the hydrophobicity of RFAC but inhibits its hydration process. The optimal SMS dosage for RFAC under dry–wet cycling is 9‰, which achieves a balance between hydrophobicity enhancement and pore structure optimization. Compared with ordinary RFAC, the specimen exhibits 12.9‰ and 17.6% increases in compressive strength and RDEM, respectively, after 30 cycles, accompanied by reductions of 25.8%, 52.7%, and 80.0% in peak free chloride content, chloride erosion depth, and convection zone depth, respectively. RFAC with 9‰ SMS exhibits a denser matrix with lower porosity and fewer corrosion products. SMS enhances chloride resistance mainly by reducing water transport and chloride ion ingress through hydrophobic modification. Dry–wet cycling gradually deteriorates the SMS-induced hydrophobic film through the weakening of Si-C-related structures, while the Si-O-Si framework remains relatively stable. A quantitative correlation between the contact angle and free chloride ion content is established, and the modified Lucas–Washburn equation provides a reasonable description of chloride ion penetration depth.

## 1. Introduction

The development of marine engineering, such as cross-sea bridges, port terminals, subsea tunnels, and offshore drilling platforms, requires an enormous consumption of cement-based materials [1,2]. Chloride salt corrosion and degradation of reinforcement represent one of the most critical issues for reinforced concrete structures in marine environments [3,4]. This problem is particularly severe in tidal zones, where high concentrations of chlorides and oxygen, coupled with alternating dry–wet cycles, significantly accelerate chloride ingress into concrete. Under such conditions, chloride transport involves not only diffusion but also convection [5,6,7,8,9,10].

To enhance the erosion resistance of reinforced concrete structures, strategies such as reducing the water-to-cement ratio, incorporating mineral admixtures, and prolonging curing time can densify the microstructure, increase chloride-binding capacity, and mitigate chloride penetration [11,12,13,14,15,16,17,18,19]. However, these approaches exhibit notable drawbacks. For instance, while reducing the water-to-cement ratio and extending the curing duration densify the microstructure and decrease pore size, they simultaneously increase capillary suction, as capillary suction is inversely correlated with pore size [20,21]. This effect facilitates the ingress of external water and aggressive ions through capillary absorption or under hydrostatic pressure via capillary pores, a phenomenon identified by many researchers as a critical factor [22,23,24]. In addition, mineral admixtures enhance the compactness of concrete by consuming calcium hydroxide and generating C-S-H gel, thereby improving chloride adsorption and binding capacity under chloride wet–dry cycles. However, at later stages of exposure, concrete incorporating mineral admixtures tends to exhibit a more distinct convection zone, a phenomenon also referred to as the skin effect [25,26,27]. Moreover, mineral admixtures reduce the alkalinity of the pore solution, which in turn lowers the chloride threshold—also known as the acceptable chloride level for reinforcement protection [19]. Cheewaket et al. [28] reported that the chloride threshold of concrete with 50% fly ash (0.1%) is only one-third of that of ordinary concrete (0.3%).

In recent years, the hydrophobic or superhydrophobic modification of cement-based materials has attracted widespread attention due to its enormous application potential [29]. Compared with surface modification using hydrophobic agents, integrally modified hydrophobic concrete exhibits multiple advantages. The former is vulnerable to high temperatures and ultraviolet radiation, which can lead to premature coating degradation; moreover, once cracks develop in the concrete, the surface hydrophobic layer becomes susceptible to failure [30,31]. The permeability of integral waterproof concrete, however, is not affected by surface abrasion. Therefore, integral waterproof concrete exhibits superior durability performance compared to concrete with surface protective layers [32]. Hydrophobic modification demonstrates remarkable effectiveness in enhancing the resistance of cement-based materials to chloride ingress. Stearic acid and calcium stearate have been confirmed to be effective in this regard. Maryoto conducted a comparative study on the chloride penetration resistance of concrete incorporating 2–2.5% calcium stearate and found that chloride penetration at a depth of 6 mm was reduced by up to 113% [33,34]. Qu et al. incorporated ground granulated blast furnace slag hydrophobically modified with stearic acid into lightweight concrete and observed that, after 63 days of immersion in NaCl solution, the chloride penetration depth decreased by approximately 90% compared to the control concrete [35]. Chen et al. investigated that the chloride migration coefficient of concrete containing 4% calcium stearate was reduced by 26% [36].

Sodium methyl silicate (SMS) is a waterproofing material with excellent crystallization permeability that has attracted widespread attention in both engineering practice and academic research in recent years. This is due to its favorable properties such as water resistance, impermeability, and anti-corrosion performance [37,38,39]. SMS can react with water and carbon dioxide to generate silanol, and it can also undergo chemical reactions with silicate materials, thereby forming an insoluble polymer compound (hydrophobic layer) on the surface and within the interior of structural materials, which prevents external moisture from penetrating into the concrete structure [38]. Guo et al. investigated the application of SMS in mitigating the corrosion of reinforced concrete induced by deicing salts and demonstrated that the salt freeze loss mass of concrete was reduced by up to 96% [39]. Chen et al. reported that when immersed for 90 days in a composite salt solution containing both sulfates and chlorides, concrete with 0.2% SMS exhibited a chloride ion content of 0.315% at a depth of 6 mm from the exposed surface, which represented a 13.93% reduction compared to concrete without sodium methyl silicate [38]. However, to date, although the effectiveness of hydrophobic modification in enhancing the corrosion resistance of concrete has been well established, and a few researchers have explored the feasibility of using SMS to improve the hydrophobicity and durability of concrete, the mechanism by which sodium methyl silicate influences concrete performance under chloride salt dry–wet cycling has not yet been reported. Moreover, the degradation mechanisms of the hydrophobic film formed in long-term service environments involving chloride dry–wet cycles remain insufficiently elucidated. Furthermore, the influence of hydrophobicity degradation on the penetration depth of chloride-bearing aggressive solutions remains unclear, which is critical for the protection of steel reinforcement in concrete.

Given the substantial annual generation of construction waste, utilizing recycled fine aggregate (RFA) from construction waste as a substitute for natural sand not only addresses the challenge of construction waste recycling but also alleviates the current shortage of river sand [40,41,42,43,44,45]. Under chloride salt dry–wet cycling, the use of RFAs increases the amount of free chloride ions in concrete due to their inherent defects, such as high porosity and low density, and it further accentuates the convection- and diffusion-dominated transport zones [46]. Applying SMS to modify the hydrophobicity of RFAC exposed to chloride dry–wet cycles constitutes an effective strategy for enhancing its durability in such environments.

Based on the prediction model for equivalent exposure time in indoor dry–wet cycling tests for concrete in marine tidal zones [47], the study adopted a methodology involving immersion in saturated sodium chloride solution combined with the air-drying method to simulate the dry–wet cycling process. A sustained four-point bending load is applied throughout the entire test duration. Hydrophobic RFAC incorporating SMS at different dosages is prepared and subjected to 30 cycles of chloride dry–wet exposure. The deterioration of mechanical properties, along with variations in contact angle, water absorption, and dehydration rate before and after cycling, is tested to evaluate changes in hydrophobicity. The chloride resistance of RFAC with different SMS dosages is assessed through measurements of free chloride ion content, erosion depth, and capillary suction height. Microstructural changes are investigated using SEM, MIP, and XRD, while XPS and FT-IR are employed to examine the chemical bonds and functional group variations induced by SMS, thereby analyzing hydrophobic durability. In addition, the erosion-resistance mechanism of SMS-modified RFAC is discussed, a quantitative relationship between the contact angle and the free chloride ion content is established, and the Lucas–Washburn equation is modified to describe the penetration characteristics under chloride salt dry–wet cycling, thereby providing a basis for the protection of steel reinforcement in concrete.

## 2. Experimental Program

### 2.1. Materials

**Figure 1:**
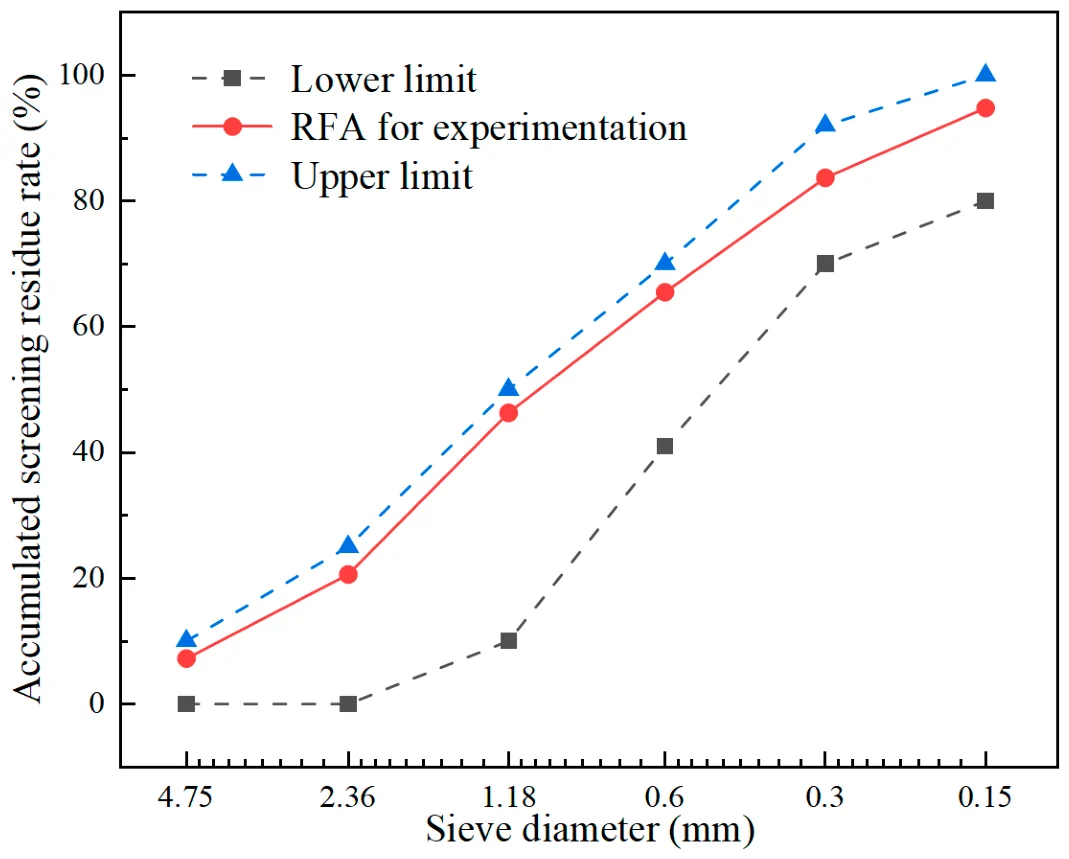
Grading curve of RFA.

The experiment employs P.O 52.5 ordinary Portland cement, Grade I fly ash (FA), S95 ground granulated blast-furnace slag (GGBS), and Grade I white silica fume (SF) with a particle size of 2.43 μm as cementitious materials, with their specific chemical compositions and physical properties listed in Table 1. The coarse aggregate consists of crushed basalt purchased from the market, with a particle size range of 4.75–25 mm, an apparent density of 2735 kg/m^3^, a crushing value of 8.6%, and a water absorption of 0.7%. The RFA is supplied by Jiangsu Lvhe Environmental Technology Co., Ltd. (Changzhou, China), with an apparent density of 2455 kg/m^3^, a crushing value of 18.1%, and a water absorption of 10.2%. The particle size distribution curve, shown in Figure 1, meets the grading requirements of Zone II specified in GB/T 25176-2010 [48]. A polycarboxylate-based water-reducing agent (SP) with a water-reducing efficiency of 21% is used to adjust the workability of the concrete. SMS solution with a solid content of 42% is adopted as the hydrophobic agent, characterized by a pH of 14, a density of 1.3 g/cm^3^ at 25 °C, and a polysiloxane content of no less than 24%.

### 2.2. Mix Proportion and Specimen Preparation

The mix proportion design for the RFAC was based on a full calculation method [49], targeting a compressive strength of C50, with a water-to-binder ratio controlled at 0.326. According to the current research [38,50], the dosage of SMS was added at 2‰ to 12‰ by mass of the cementitious materials. Therefore, in this study, four different dosages of SMS, namely 3‰, 6‰, 9‰ and 12‰ (by mass of the cementitious materials), were selected, and the corresponding concrete specimens were designated as S3, S6, S9, and S12, respectively. RFAC without SMS addition was used as the control group, designated as S0. For convenience in applying loads during the dry–wet cycling process, all specimens were cast with dimensions of 100 mm × 100 mm × 400 mm. The specimens were prepared using a two-step mixing method and then placed in molds. After standard curing for 24 h, the specimens were demolded and transferred to a curing chamber (temperature 20 ± 2 °C, relative humidity ≥ 95%) for 28 days prior to dry–wet cycling. The detailed mix proportions are presented in Table 2.

### 2.3. Dry–Wet Cycling Test

**Figure 2:**
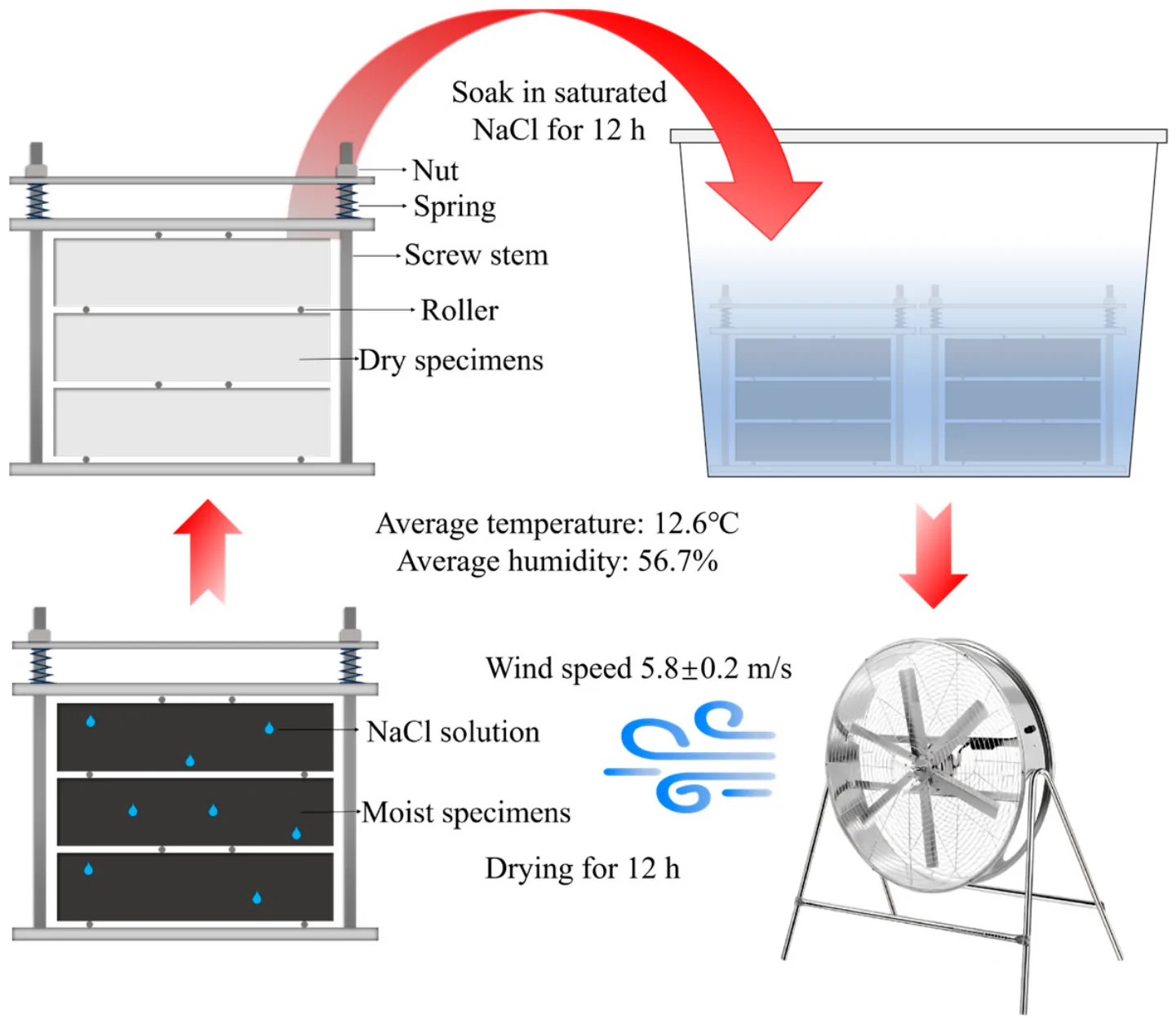
Experimental process chart.

The dry–wet cycling environment in this study was designed with reference to previous research [46,47,51,52], where specimens were subjected to sustained four-point bending under a load corresponding to 25% of their flexural strength. During the wetting phase, the entire loading setup was immersed in saturated sodium chloride solution to accelerate chloride ion ingress under simulated marine environmental conditions. In the drying phase, an industrial fan was used to impose an air velocity of 5.95 m/s on the specimens, which was determined with reference to the average annual wind speeds of major coastal cities in China over the past five years [53]. The wet-to-dry time ratio was set at 3:1, i.e., 12 h of immersion followed by 36 h of drying, which reflects the characteristics of many coastal environments. In addition, to guarantee one-dimensional chloride ion ingress, the four lateral faces of the specimens were sealed with epoxy resin, leaving only one pair of opposite surfaces (400 mm × 100 mm) exposed. A total of 30 cycles were carried out, and the number of dry–wet cycles was equivalently correlated with service time using the prediction model for equivalent exposure time in indoor dry–wet cycling tests for concrete in marine tidal zones proposed by Chen et al. [47]. During the cycling process, the recorded average temperature was 12.6°C and the average relative humidity was 56.7%, corresponding to an equivalent service life of 78.9 years [47]. The experimental procedure is illustrated in Figure 2.

### 2.4. Test Methods

#### 2.4.1. Compressive Strength and Relative Dynamic Elastic Modulus

**Figure 3:**
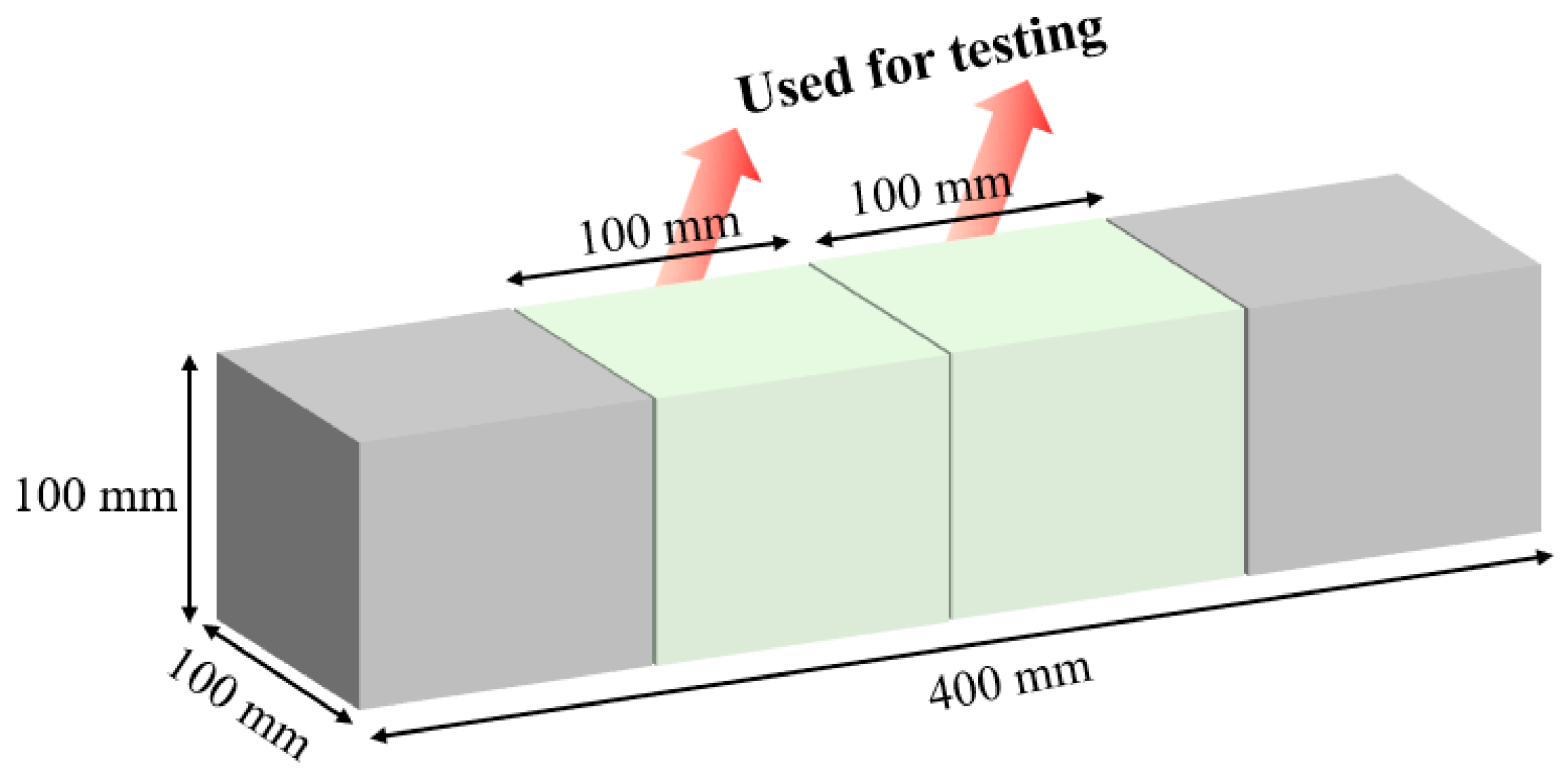
Specimens used for testing during dry–wet cycles [55].

The compressive strength was evaluated using cubic specimens with a side length of 100 mm in accordance with GB/T 50081-2019 [54]. These cubic specimens were obtained by cutting rectangular prisms measuring 100 mm × 100 mm × 400 mm, with the two central portions selected for testing, as illustrated by the blue sections in Figure 3. Each test involved three specimens, and their average value was adopted as the representative compressive strength. The variation rate of compressive strength was calculated using the following formula:

(1) $$\Delta f_{\mathrm{ck}}=\frac{f_{\mathrm{ck}}-f_{c0}}{f_{c0}}$$

where $\Delta f_{\mathrm{ck}}$ is the change rate of compressive strength, %; $f_{\mathrm{ck}}$ is the compressive strength of the specimen after the k-th cycle, MPa; and $f_{c0}$ is the strength prior to the chloride salt dry–wet cycling, MPa.

The resonant frequency of the rectangular prism specimens was tested using a DT-20 concrete relative dynamic elastic modulus tester (Changzhou Lucky Electronic Equipment Co., Ltd., Changzhou, China), and the relative dynamic elastic modulus (RDEM) was calculated accordingly.

#### 2.4.2. Chloride Content

**Figure 4:**
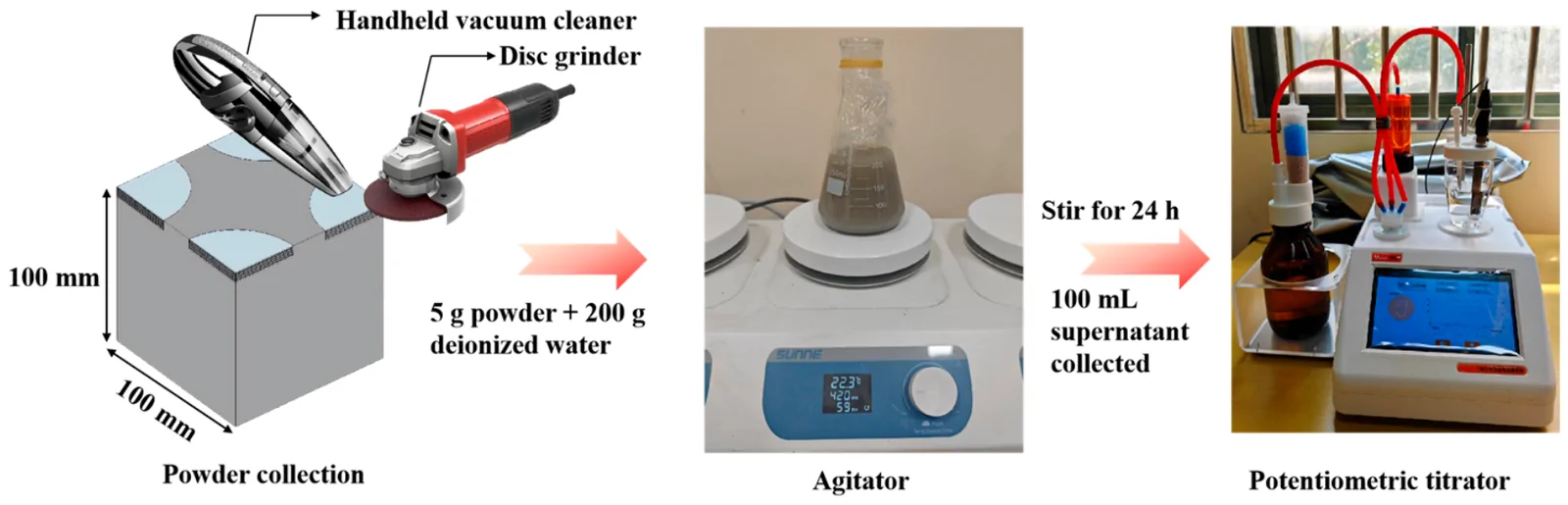
Procedure for measuring free chloride ion content.

The epoxy resin on the concrete surface was removed to avoid any interference with the results. Subsequently, a handheld angle grinder equipped with a vacuum collector (Cangzhou Hecheng Environmental Protection Equipment Co., Ltd., Cangzhou, China) was used to collect the powder from 1 to 30 mm beneath the exposed surface. To characterize the convection-affected region, powder samples were collected at 1 mm intervals within the first 10 mm. A 5 g powder sample was mixed with 200 g deionized water and stirred for 24 h to ensure full leaching of free chloride ions. Then, 100 mL of the supernatant was collected and analyzed for free chloride ion content using a T860 fully automatic potentiometric titrator (Hanon Advanced Technology Group Co., Ltd., Jinan, China). The detailed procedure is shown in Figure 4.

#### 2.4.3. Chloride Ion Erosion Depth and Capillary Water-Absorption Height

**Figure 5:**
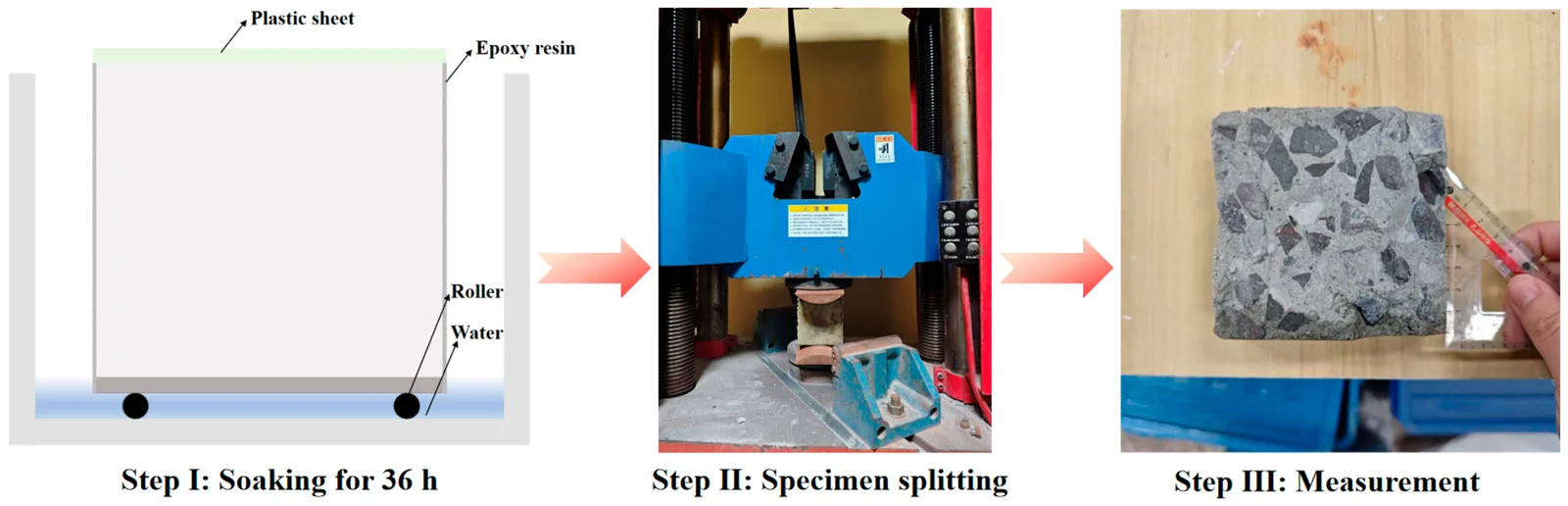
Procedure for measuring capillary water absorption height.

The capillary water absorption height was measured with reference to ASTM C1585-13 [56], using specimens taken from the drying stage of the dry–wet cycling. Epoxy resin was reapplied to the cut surfaces to expose only the testing face, and the top surface was covered with a plastic sheet. Each specimen was placed on rollers with its bottom immersed 3 mm below the water level. To align with the erosion test, the absorption time was set equal to the soaking duration in the dry–wet cycles. After 36 h, the specimen was split, and the capillary absorption height was measured on the fractured surface. The detailed procedure is shown in Figure 5.

**Figure 6:**
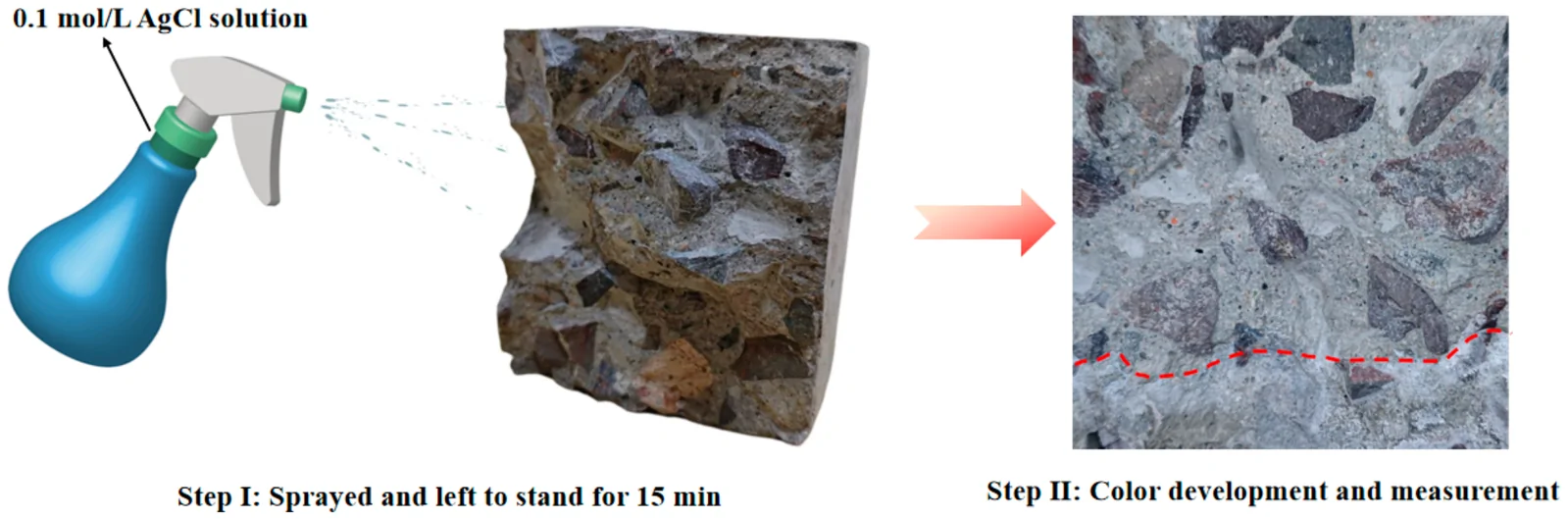
Procedure for measuring chloride ion erosion depth.

The chloride ion erosion depth was determined in accordance with GB/T 50082-2009 [57]. After every five dry–wet cycles, each specimen was split along its mid-section using a compression testing machine. A 0.1 mol/L AgCl solution was uniformly sprayed onto the freshly fractured surface, and the specimen was left undisturbed for 15 min, after which the length of the whitened region was measured. The detailed procedure is shown in Figure 6.

To minimize measurement errors, the outermost 10 mm of the fractured surface and areas containing coarse aggregates were excluded from the assessment. A total of ten measurements were taken, and their average value was used as the final result.

#### 2.4.4. Contact Angle

Fragments were taken from the exposed surface of the specimens, and residual salt crystals on the surface were rinsed off with deionized water. Subsequently, at room temperature, the contact angle between water droplets and the hardened paste surface was measured using a contact angle goniometer (JY-82B, Kruss DSA, Hamburg, Germany) by means of the sessile drop method combined with the ellipse-fitting approach. The contact angle was automatically determined by the tangent method at the three-phase contact line of the droplet image after 1 s of contact between the water droplet and the specimen surface, corresponding to the angle between the liquid–vapor interface and the solid surface.

#### 2.4.5. Water Absorption and Dynamic Water Desorption

According to GB/T 50081-2019, the water absorption of the specimens was measured after 0, 5, 15, 20, 25, and 30 cycles. Upon completion of the 15th and 30th immersion cycles, the dehydration rate of the specimens was characterized by monitoring the mass variation during the air-drying process.

To ensure the reliability and repeatability of the experimental results, all mechanical and durability tests were conducted using parallel specimens. For each mixture, three specimens were prepared for compressive strength, dynamic elastic modulus, contact angle, capillary absorption, and chloride penetration tests. The reported values represent the average results obtained from repeated measurements. The experimental deviations among parallel specimens were within an acceptable range, indicating good repeatability of the testing procedures. Each experiment was conducted following the corresponding standard procedures, and repeated measurements were performed to ensure the reliability and reproducibility of the experimental results.

#### 2.4.6. Microstructure

The microstructure of RFAC before and after chloride salt dry–wet cycles was analyzed using FE-SEM (SUPRA55, Zeiss, Oberkochen, Germany), focusing on the ITZ characteristics, pore morphology, and microcrack evolution. XRD (Rigaku, Akishima, Japan) was employed to identify the phase composition and mineralogical changes before and after cycling. The pore structure after 30 dry–wet cycles was evaluated using MIP (AutoPore V 9600, Micromeritics, Norcross, GA, USA). XPS (Thermo Fisher Scientific, Waltham, MA, USA) and FTIR (IS50, Thermo Fisher Scientific, Waltham, MA, USA) were used to investigate the changes in chemical bonds and functional groups associated with hydrophobic film degradation.

## 3. Result Analysis

### 3.1. Compressive Strength

**Figure 7:**
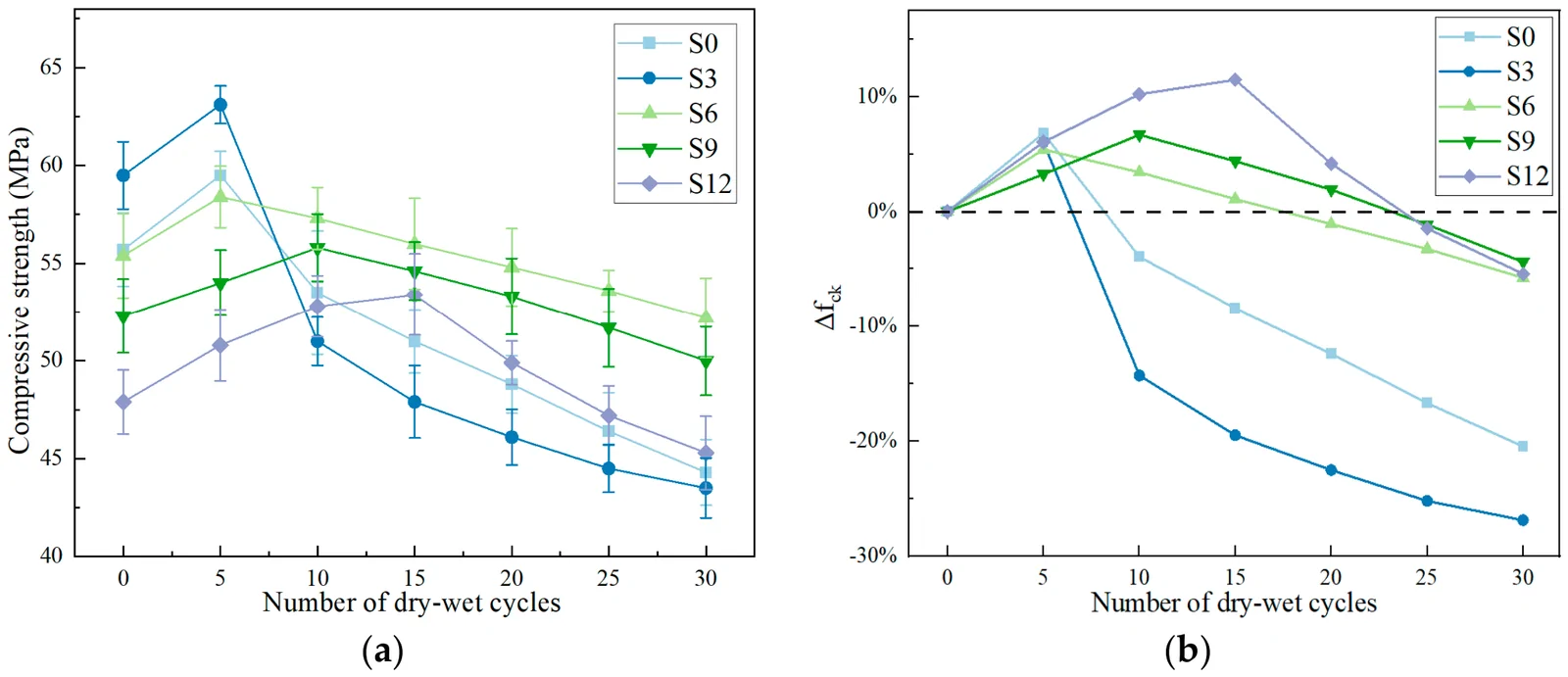
Compressive strength (**a**) and change rate curves (**b**) under different dry–wet cycles.

The variation in compressive strength of RFAC with different dosages of SMS under wet–dry cycling is presented in Figure 7. With the increase in SMS content, the initial compressive strength of RFAC first increases and then decreases. The S3 mixture exhibits the highest initial compressive strength, which is 6.8% higher than that of the control group S0, indicating that an appropriate dosage of SMS contributes to strength enhancement. As an alkaline compound, SMS functions as an alkaline activator, promoting the formation of greater quantities of C-S-H and C-A-S-H from supplementary cementitious materials such as fly ash, silica fume, and GGBS [58,59]. However, when the SMS dosage exceeds 3‰, the compressive strength begins to decline. For instance, the lowest initial strength is observed in S12, with a value of 47.9 MPa, 4.0% lower than S0 and below the designed target strength. This is because the adverse effects of hydrophobicity surpass the previously mentioned beneficial effects. The hydrolysis of SMS generates methylsilanol groups that anchor onto the surfaces of cementitious phases and aggregates, imparting hydrophobicity. This hydrophobic layer impedes water ingress, thereby limiting the availability of moisture necessary for continued hydration reactions, resulting in a retardation of both the rate and extent of hydration [60,61]. Additionally, the methyl groups on the outer side of the methylsilanol units introduce electrostatic repulsion during the condensation process, which hinders the approach of other polar monomers and obstructs the formation of Si-O bonds in C-S-H and C-A-S-H structures [58]. The enhanced compressive strength of SMS-modified RFAC can be attributed to the combined effects of hydration product evolution, pore structure refinement, and improved interfacial characteristics. The XRD and FTIR results indicate that SMS incorporation changes the characteristics of hydration-related phases, suggesting that SMS may influence the hydration evolution of the cementitious matrix. Previous studies have reported that silicate-containing additives can participate in the modification of cement hydration products and contribute to the development of C–S–H-related phases. Meanwhile, the refined pore structure observed from MIP analysis indicates that SMS contributes to matrix densification and reduces the defects within the cement paste and interfacial transition zones. Therefore, the strength enhancement is considered to result from the synergistic effects of hydration evolution, pore refinement, and interface improvement.

As the number of dry–wet cycles increases, the compressive strength follows a rising-then-falling trend. The initial strength gain is attributed to pore-filling effects from salt crystallization residues and reaction products of chloride ions, such as Friedel’s salt and expansive chlorides. However, as these products excessively accumulate, internal expansion stress develops, leading to cracking and spalling of the concrete [62,63,64]. Interestingly, SMS appears to enhance the durability of RFAC by delaying the onset of compressive strength degradation. For instance, the strength of S9 begins to decline only after 10 cycles, whereas S12 shows strength loss only after 15 cycles.

Due to immersion in saturated chloride solution and the application of four-point bending load, the compressive strength of the specimens decreases markedly, which is consistent with our previous findings [52]. After 30 chloride salt dry–wet cycles, the residual compressive strength relative to the initial value decreases by 20.5%, 26.9‰, 5.8%, 4.4%, and 5.4% for S0, S3, S6, S9, and S12, respectively. In the case of S3, the presence of SMS does not sufficiently densify the microstructure due to inadequate hydration, and its relatively weak hydrophobicity fails to prevent moisture ingress during wetting or facilitate moisture removal during drying. As a result, chloride ions accumulate internally, and salt crystallization stress becomes the dominant damage mechanism [65]. In contrast, S6, S9, and S12 benefit from their enhanced hydrophobicity, which effectively mitigates strength loss during cyclic exposure, even though their initial compressive strengths are not particularly high.

### 3.2. RDEM

**Figure 8:**
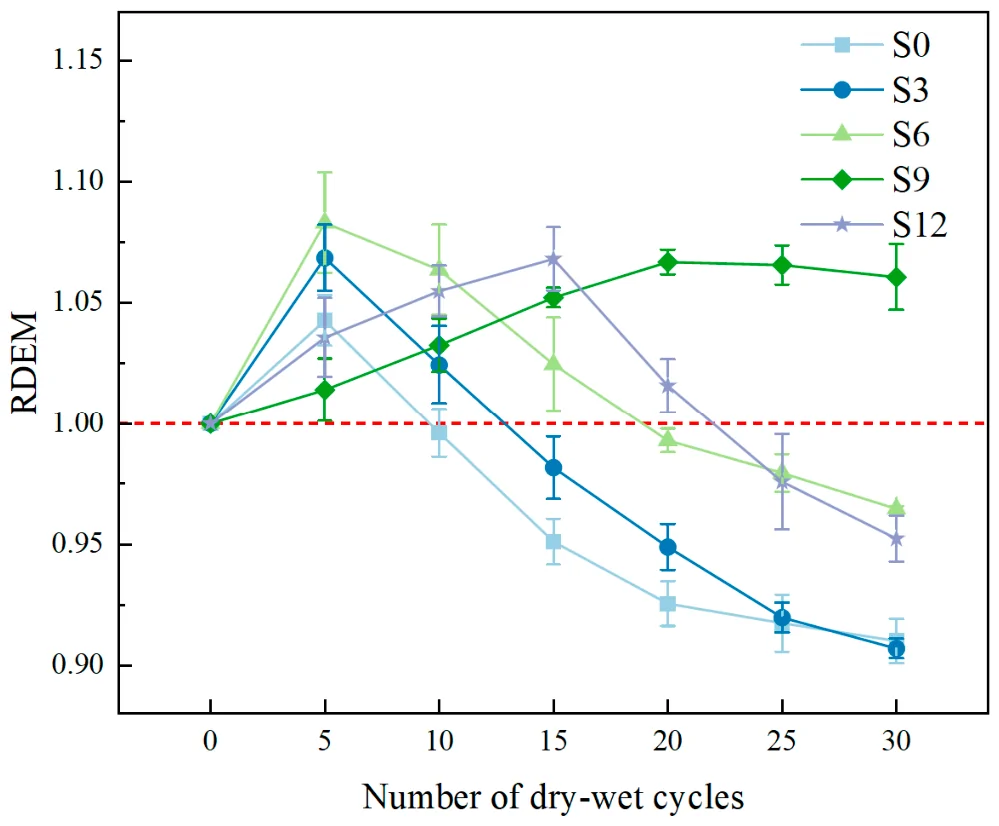
RDEM of RFAC with different dosages of SMS during dry–wet cycles.

The RDEM was measured to characterize the evolution of dynamic stiffness and damage degree of RFAC under dry–wet cycling, as illustrated in Figure 8. With increasing cycle numbers, the RDEM values exhibited an initial increase followed by a subsequent decrease. After 5 cycles, the RDEM values of S0 to S12 increased to 1.04, 1.07, 1.08, 1.01, and 1.03 times their initial values, respectively. The initial increase in RDEM is mainly attributed to the temporary pore-filling effect of salt crystals generated during the drying process, which improved the resistance of the matrix to dynamic loading, particularly in the tensile zone under four-point bending [52]. In addition, the precipitation of chloride-related crystalline products, such as Friedel’s salt (3CaO·Al_2_O_3_·CaCl_2_·10H_2_O), within the outer region of specimens may also contribute to the increased stiffness by partially filling internal pores [19,55,63]. With further dry–wet cycling, excessive accumulation of salt crystals and chloride-binding products within the pores generated crystallization pressure on the pore walls, promoting the initiation and propagation of microcracks and consequently reducing the stiffness of the specimens [62,66]. The RDEM values of S0–S6 began to decrease after 5 cycles, whereas S12 maintained an increasing trend until 15 cycles, and S9 showed a decrease after 20 cycles. These results indicate that SMS incorporation can delay the deterioration of RFAC during chloride salt dry–wet cycling. On the one hand, SMS introduces hydrophobic characteristics into the concrete matrix, reducing capillary absorption of chloride-containing solutions and limiting chloride accumulation within the pores. Consequently, the crystallization pressure induced by salt deposition and chloride-related products is reduced, delaying damage development. On the other hand, excessive SMS incorporation may restrict water migration during hydration, resulting in increased porosity and weaker interfacial structures compared with the control mixture (S0) [67,68,69]. The increased pore volume may provide additional space for salt crystallization and corrosion products, thereby partially delaying crack initiation. However, excessive pore connectivity can also facilitate the transport of aggressive ions and accelerate long-term deterioration.

The enhanced resistance of SMS-modified RFAC against chloride-induced deterioration is therefore mainly associated with reduced water and chloride transport caused by hydrophobic modification, together with the favorable pore structure obtained at an appropriate SMS dosage. Previous studies have reported that silane/silicate-based additives can improve the durability of cementitious materials by reducing surface wettability and modifying pore characteristics. Accordingly, the improved durability of SMS-modified RFAC is attributed to the synergistic contributions of hydrophobic protection and microstructural regulation rather than a single mechanism.

At the end of the dry–wet cycling, only S9 retained an RDEM value higher than its initial value (1.06), whereas all other mixtures exhibited different degrees of reduction. At this stage, the adverse effects associated with salt crystallization and microcrack development exceeded the beneficial pore-filling effect. Compared with S0, S6 and S12 exhibited 6.0% and 4.6% higher RDEM values, respectively, whereas S3 showed a 3.4% reduction. The relatively lower improvement of S3 may be related to the insufficient hydrophobic protection provided by the lower SMS dosage. Although S12 showed a delayed decrease in RDEM due to its larger pore volume accommodating salt accumulation, its excessive pore connectivity and weakened microstructure may adversely affect long-term durability. Therefore, an appropriate SMS dosage is essential to achieve a balance between hydrophobic protection and structural integrity.

### 3.3. Hydrophobic Performance

#### 3.3.1. Wettability

**Figure 9:**
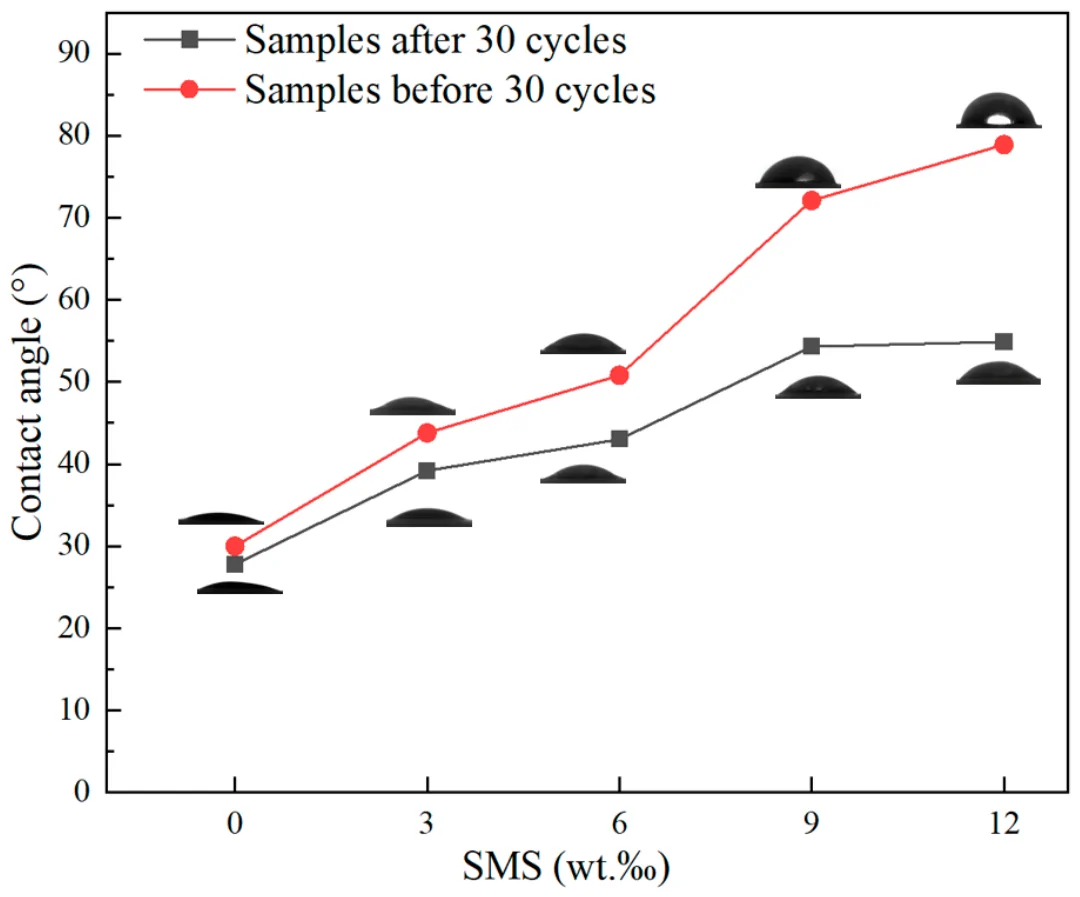
Wettability of RFACs with various SMS additions.

Figure 9 shows the changes in surface contact angles of different RFAC specimens before and after 30 wet-dry cycles as the SMS content increases. The surface contact angles of different specimens are measured to evaluate the effect of SMS on the hydrophobicity of RFAC. In the absence of SMS, water rapidly penetrates into the interior of the S0 specimen, resulting in a fully wetted surface. With an increasing SMS dosage, the contact angle also increases, exhibiting a rapid–slow growth trend. When the dosage is 3‰, the change in contact angle is negligible; at 9‰, the contact angle sharply increases to 72.1°. Further increases in SMS dosage lead to only a slight increase, with the S12 specimen reaching 78.9°. SMS undergoes dehydration–condensation under the action of water and CO_2_, forming a network-like hydrophobic layer on the surface of hydration products, thereby increasing the contact angle. As hydrophobicity improves, capillary water transport within the matrix becomes more difficult; consequently, even in the presence of sufficient methylsilanol and CO_2_, the lack of water limits further increases in the contact angle.

Under chloride salt dry–wet cycling, the contact angles of all specimens show a decreasing trend, indicating deterioration in RFAC hydrophobicity. The greater the initial contact angle, the larger the reduction. For instance, the contact angle of the S12 specimen decreases to 54.9°, representing a 30.4% reduction and approaching that of S9 (54.3°). This reduction is attributed to partial failure of the hydrophobic layer as well as the accumulation of salt crystals and corrosion products on the matrix surface. These deposits markedly increase the surface free energy, causing the solid–liquid interfacial adhesion to exceed the liquid cohesive force, thus rendering the specimens more hydrophilic [70,71].

#### 3.3.2. Water Absorption

**Figure 10:**
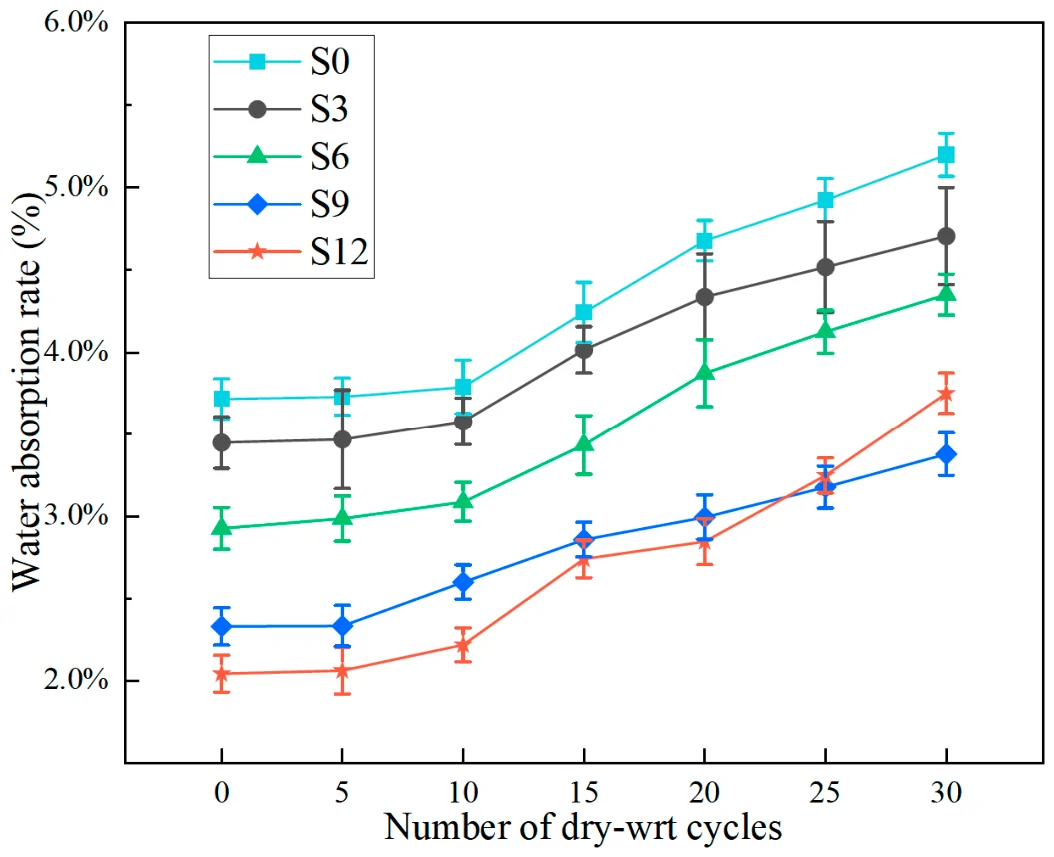
Water absorption of RFACs with various SMS additions undergoing wet–dry cycling.

The variation in water absorption of RFAC with increasing numbers of dry–wet cycles is shown in Figure 10. As a multi-porous hydrophilic material, concrete inherently exhibits high water absorption, with the initial water absorption of S0 reaching 3.7%. The incorporation of SMS effectively reduces the water absorption of RFAC; compared with S0, the absorption rates of S3–S12 are reduced by 7.2%, 21.3%, 37.3%, and 45.0%, respectively. This reduction is mainly attributed to the enhanced surface hydrophobicity induced by SMS. Although SMS slightly reduces the compactness of RFAC, it significantly inhibits moisture transport within the matrix. The reduced wettability prevents capillary pores from reaching full saturation, thereby limiting effective water absorption and reducing the contribution of connected pore channels to water transport.

With prolonged exposure to chloride salt dry–wet cycling, the protective effect of SMS gradually changes due to the combined influence of salt attack, cyclic moisture variation, and microstructural deterioration. As shown in Figure 10, the water absorption of all specimens increases with increasing cycle numbers, which is mainly associated with the generation and propagation of microcracks and the gradual deterioration of pore structures during cycling. Among all mixtures, S12 exhibits a pronounced increase in water absorption after 15 cycles, which may be related to the weakened hydrophobic protection and the expansion of pores and cracks caused by excessive SMS incorporation. However, because the deterioration mainly occurs in the surface region exposed to external attack, the internal matrix still maintains relatively favorable hydrophobic characteristics, resulting in a lower water absorption than S0. After 30 cycles, S9 exhibits the lowest water absorption, with a reduction of 35.0% compared to S0. This behavior indicates that an appropriate SMS dosage can achieve a favorable balance between hydrophobic protection and pore structure stability, thereby effectively suppressing water transport during long-term chloride salt exposure.

#### 3.3.3. Dynamic Water Desorption

**Figure 11:**
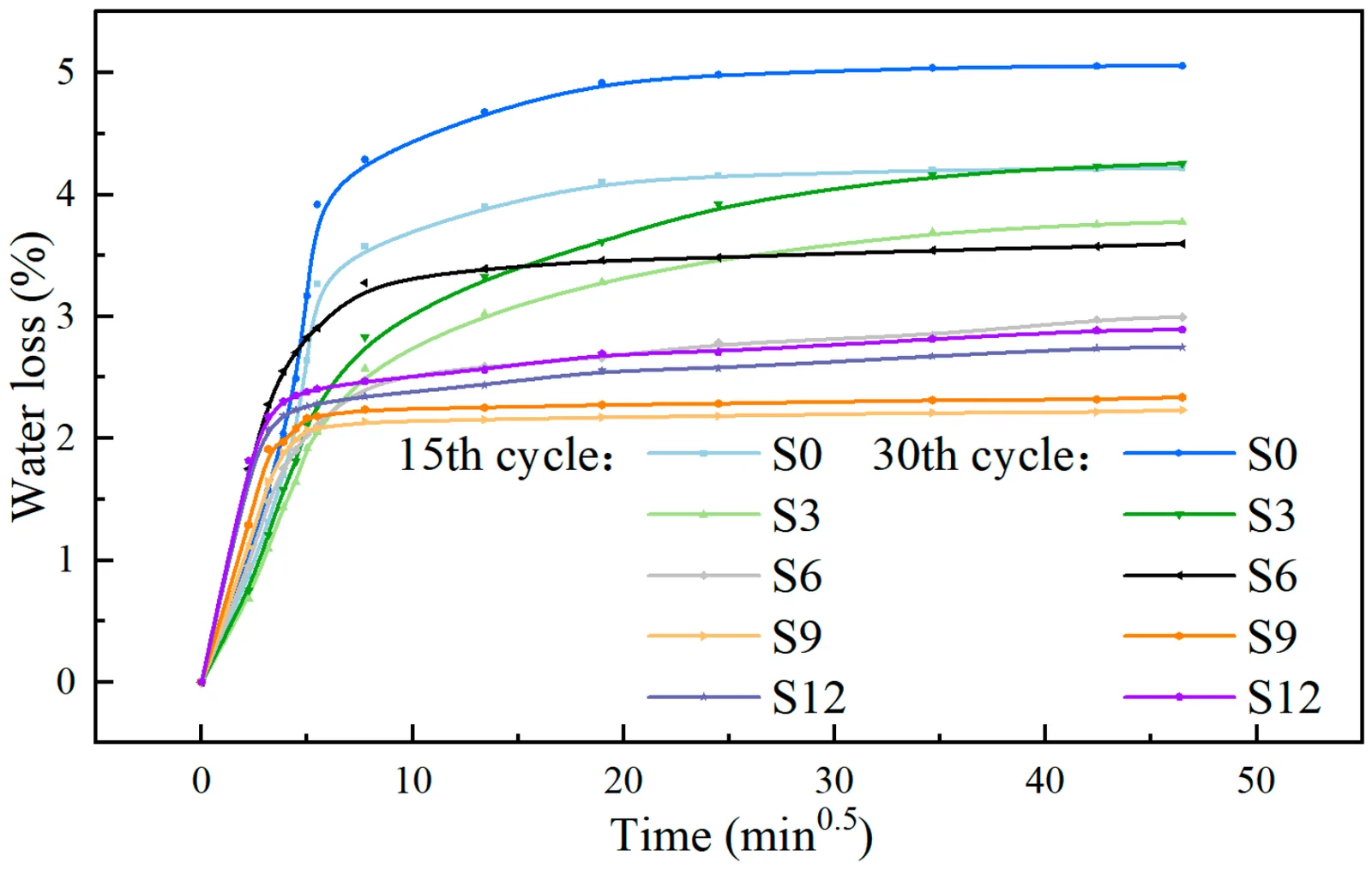
Dynamic water desorption of RFACs with various SMS additions.

The moisture loss of the specimens under air-drying conditions is shown in Figure 11. Within the initial 25 min, the surfaces of the specimens undergo rapid dehydration under the influence of a fan. The presence of SMS further expedites this process by increasing the contact angle, enabling the surface to shed water droplets more easily, analogous to the lotus-leaf effect. After 25 min, however, SMS significantly reduces the rate of moisture loss. At the end of drying after the 15th cycle, the dehydration rates of S3–S12 are 10.4%, 28.9‰, 47.0%, and 34.38% lower, respectively, than that of S0. After the 30th cycle, the corresponding decreases are 15.9‰, 28.9‰, 53.8%, and 42.8%, respectively. The moisture loss beyond 25 min is primarily attributed to the migration of capillary water from the specimen interior to the surface. Evidently, the increased contact angle induced by SMS inhibits this migration, allowing the internal matrix to maintain a relatively stable moisture content.

For all specimens, the final mass loss rate after 30 cycles is higher than that after 15 cycles, mainly due to the extension and enlargement of capillary pores and the formation of microcracks induced by chloride salt attack, which facilitate moisture migration. In addition, as illustrated in Figure 9, the contact angle decreases during the later stages of cycling, representing a significant contributing factor that facilitates the easier escape of internal moisture through the capillary pores.

#### 3.3.4. Appearance

**Figure 12:**
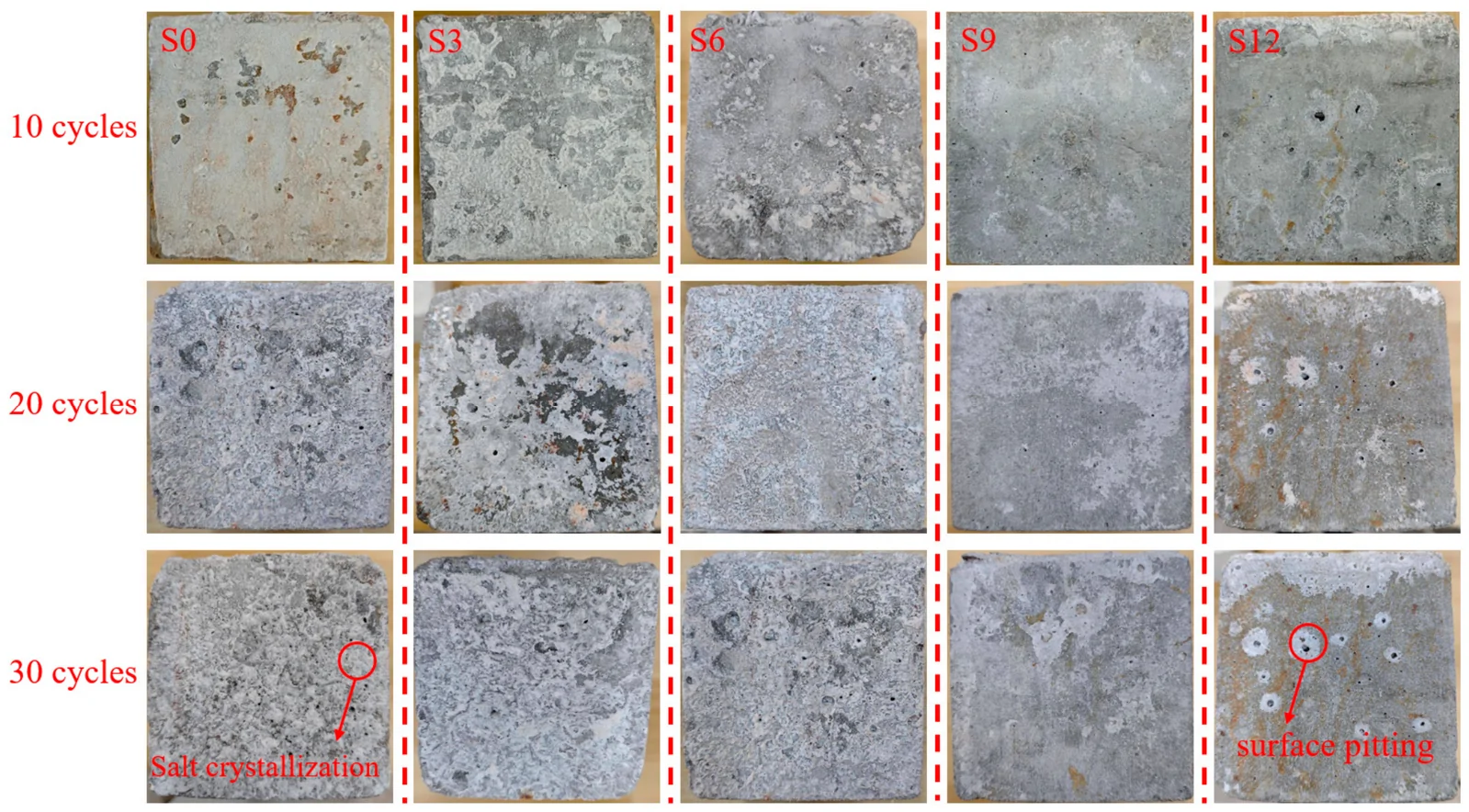
Appearance of RFACs with various SMS additions undergoing dry–wet cycling.

The apparent morphology of the specimens after the 10th, 20th, and 30th cycles is shown in Figure 12. The incorporation of SMS significantly enhances the surface self-cleaning ability of the specimens. Specifically, during the air-drying stage, the erosion solution on the surface and in the near-surface region is rapidly removed under the combined effects of airflow and gravity, analogous to the rolling-off of water droplets on a lotus leaf, thereby inhibiting solution accumulation on the surface and substantially reducing surface salt crystallization. For example, distinct salt crystallization is already observed on the surfaces of S0 and S3 after 10 cycles, whereas S9 and S12 still exhibit no obvious crystallization even after 30 cycles. However, with further increases in SMS content, the likelihood of sufficient contact between water and cementitious particles diminishes, leading to insufficient densification of the matrix [72]. Consequently, salt crystals and compounds such as 3CaO·CaCl_2_·15H_2_O accumulate within the capillary pores. This exerts expansive pressure on the pore walls, further increasing the pore diameter and ultimately leading to pronounced surface pitting, a phenomenon that ought to be avoided [73]. S9 exhibits a favorable balance between hydrophobicity and matrix densification, thereby providing considerable resistance to chloride salt deterioration.

### 3.4. Free Chloride Content

The free chloride ion content of the specimens at different depths under varying numbers of dry–wet cycles is shown in Figure 7. With increasing cycles, the free chloride content at all depths gradually increases, whereas the incorporation of SMS effectively reduces the free chloride ion concentration. The elevated surface contact angle induced by SMS facilitates the rolling-off of chloride-containing solution droplets from the specimen surface, thereby inhibiting surface accumulation and markedly decreasing salt crystallization (Figure 12). Consequently, diffusion driven by chloride concentration gradients is substantially weakened [74]. In addition, SMS introduces hydroxyl groups that increase the alkalinity of the matrix, thereby enhancing chloride binding and further lowering the free chloride ion content [19,75]. Most importantly, SMS reduces the capillary absorption of the matrix, which is regarded as the dominant mechanism for chloride ingress under dry–wet cycling conditions. Through dehydration and condensation, SMS forms a silane-based network film on the matrix surface, in which surface methyl groups hinder the migration of water within the pores. Consequently, the aggressive solution is largely confined to the outer surface layer, which remains at a high degree of saturation and relative humidity throughout the cycles. This reduces the adsorption capacity of the capillary pores, limits the penetration depth of the solution, and decreases the chloride concentration within the pore solution [39,76].

**Figure 13:**
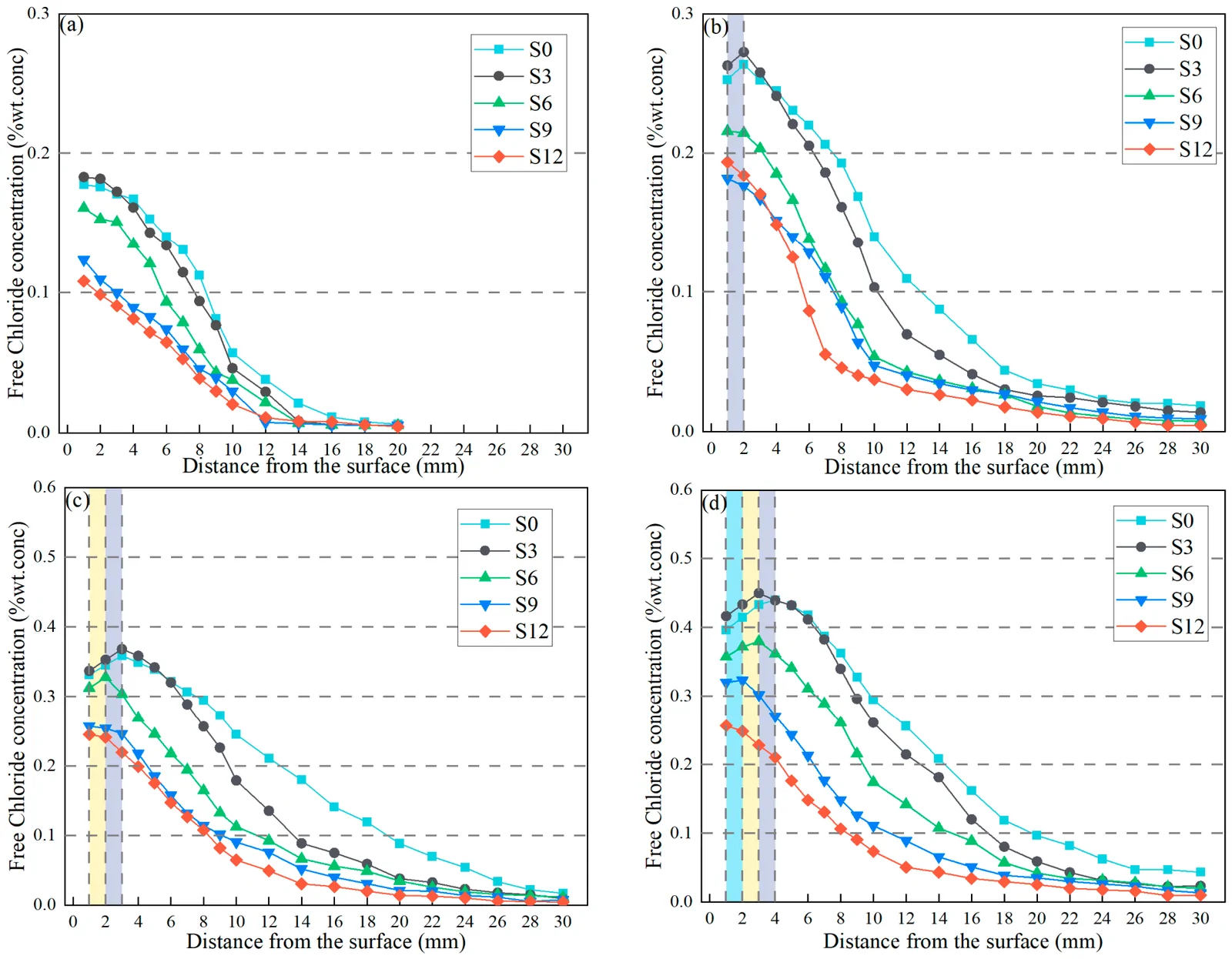

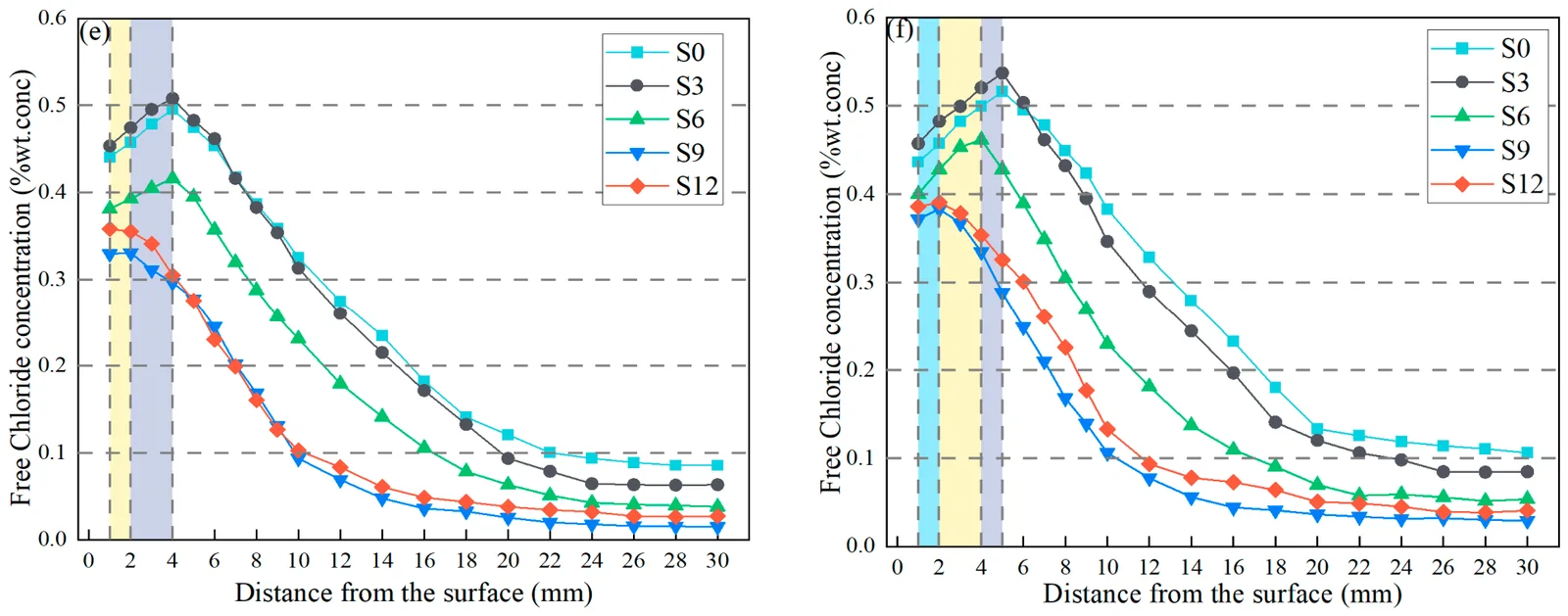
The distribution of free chloride content after different dry–wet cycles: (**a**) 5 cycles; (**b**) 10 cycles; (**c**) 15 cycles; (**d**) 20 cycles; (**e**) 25 cycles; (**f**) 30 cycles.

As the number of dry–wet cycles increases, convection zones gradually develop within specimens. As shown in Figure 13b, S0 and S3 exhibit convection zones after 10 cycles, followed by S6 after 15 cycles, as shown in Figure 13c, with the penetration depth increasing progressively with the number of cycles. In contrast, S9 and S12 exhibit convection zones only after 30 cycles, as shown in Figure 13f. Usually, chloride ions enter the matrix pores with the penetrating solution, and during the subsequent drying phase, water evaporates while chlorides remain within the pores. In the next wetting stage, additional solution transport carries chlorides further inward, thereby advancing the convection zone. Evidently, SMS suppresses this process. After 30 cycles, S9 exhibits the most favorable performance, with the peak free chloride ion content reduced by 34.7%, 40.2%, 20.5%, and 19.7% compared with S0, S3, S6, and S12, respectively, as indicated in Figure 13f, and by 72.3%, 65.3%, 45.3%, and 27.8% at a depth of 30 mm. Notably, S12 maintains the lowest free chloride ion content before the 25th cycle but exceeds that of S9 thereafter, as the excessive incorporation of SMS increases the porosity and pore size of the concrete, facilitating CO_2_ ingress. The carbonation-induced release of bound chlorides further elevates the free chloride concentration [77,78,79,80]. Therefore, excessive incorporation of SMS is undesirable. When the amount of SMS reaches a certain level, the amount of SMS does not have a direct proportional relationship with the water repellency of the concrete.

### 3.5. Capillary Rise and Chloride Ingress

**Figure 14:**
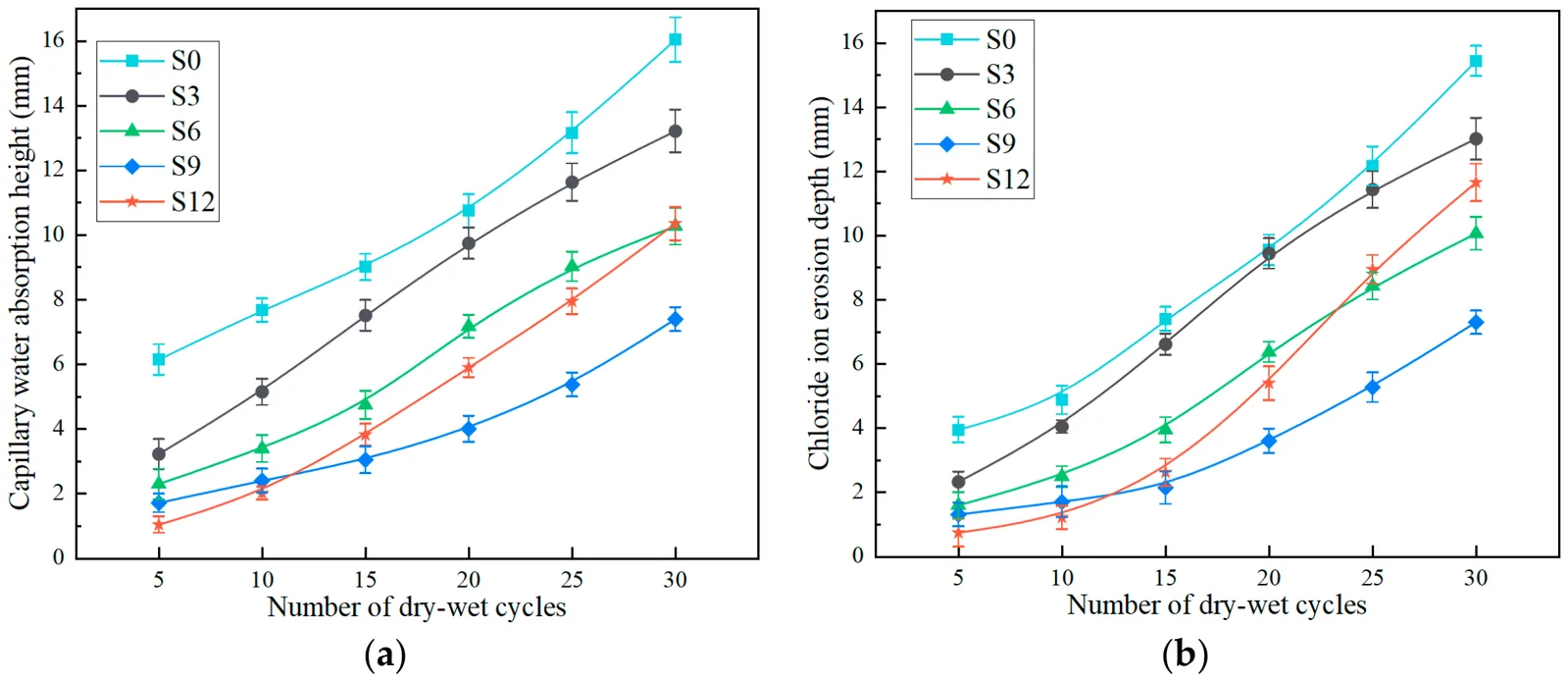
Capillary water absorption height (**a**) and chloride ion erosion depth (**b**) under different dry–wet cycles.

Figure 14 shows the changes in capillary water absorption height and chloride ion penetration depth of specimens with different SMS contents as the number of wet-dry cycles increases. SMS significantly reduces the capillary water absorption height and chloride erosion depth of RFAC at different cycles of dry–wet cycling. After 30 cycles, the capillary water absorption height of S3–S12 decreases by 17.6%, 36.0%, 53.9‰, and 35.4%, respectively, compared with that of S0, while the corresponding erosion depth decreases by 15.7%, 34.8%, 52.7%, and 24.5%. The incorporation of SMS reduces the surface free energy of the matrix, leading to an increase in the pore contact angle, which weakens capillary suction and reduces the driving force for water ingress, thereby decreasing the capillary water absorption height. The reduction in erosion depth results from the decrease in capillary water absorption height, as the siloxane network film hinders not only water penetration but also the transport of chloride ions dissolved in water. With increasing numbers of dry–wet cycles, both the capillary water absorption height and erosion depth of all specimens gradually increase. The increase in capillary water absorption height is mainly attributed to further pore deterioration, including the formation of microcracks, the development of ink-bottle pores, the transformation of closed pores into connected pores, and the reduction in contact angle. In addition to these factors, the increase in erosion depth is also associated with chloride ion accumulation, which induces the skin effect and enhances diffusion-driven transport.

To further investigate the erosion–penetration characteristics of RFAC, the difference (ΔH) between the capillary water absorption height and the erosion depth is examined, as summarized in Table 3. Under the first 10 cycles, ΔH gradually decreases with increasing SMS dosage. This is attributed to the -CH_3_ groups formed on the RFAC surface by SMS, which isolate chloride ions in the pore solution from the matrix; consequently, chloride ions are unable to form binding products with C-S-H, Ca(OH)_2_, etc., and instead remain predominantly in the free ion state. This effect becomes more pronounced with increasing hydrophobicity. As the number of cycles increases, ΔH first increases and then decreases. An increase in ΔH indicates that water penetrates to greater depths while chloride ions are retained by binding reactions or pore blocking; subsequently, water during the next wetting stage transports chloride ions to deeper regions, which is a typical feature of convection-controlled transport. When ΔH decreases and approaches zero, convection weakens; at this stage, micropores are filled with binding products and salt crystals, the chloride ion concentration increases, and diffusion becomes significant. Accordingly, the erosion mechanism of S0 shifts from convection-dominated to diffusion-dominated, whereas hydrophobic RFAC is predominantly governed by diffusion. Notably, for S12, ΔH < 0 is observed during 25–30 cycles, which may be attributed to its insufficient degree of hydration. The excessively large pores accommodate substantial amounts of salt crystals and binding products, and the elevated chloride ion concentration leads to severe diffusion and chloride release, resulting in the presence of chloride ions in regions that are not reached by water.

### 3.6. Microstructure

#### 3.6.1. SEM

**Figure 15:**
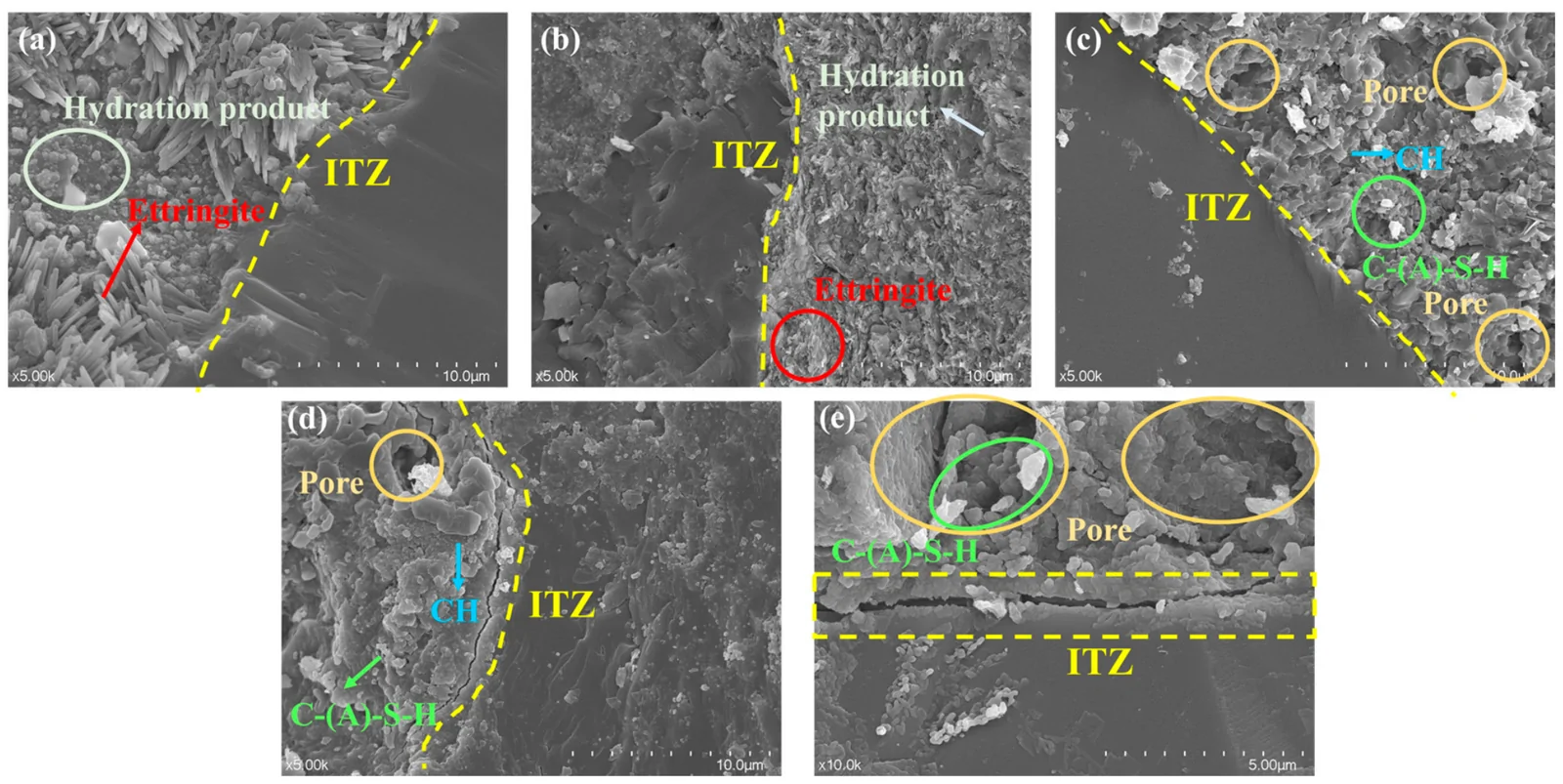
SEM images of ITZs before dry–wet cycling: (**a**) S0; (**b**) S3; (**c**) S6; (**d**) S9; (**e**) S12. ((**a**)–(**d**) scale bar 10.0 µm; (**e**) scale bar 5.00 µm).

The microstructure of the ITZ between cement paste and coarse aggregate at a curing age of 28 days is investigated by SEM, as shown in Figure 15. The incorporation of SMS into RFAC introduces a large amount of hydroxyl ions, leading to an accelerated dissolution rate of mineral phases. As a result, silicate species are more stably maintained in the pore solution, providing additional nucleation sites for C-S-H. With the progression of hydration, Ca^2+^ is continuously released and combines with hydroxyl ions to form plate-like portlandite (CH), which, together with gel-like calcium silicate hydrate (C-S-H) and hydrated calcium silicate aluminate (C-A-S-H), contributes to the dense matrix and ITZ observed in S3 and S6. This accounts for the favorable mechanical performance of S3 and S6 prior to exposure to cyclic conditions. In addition, a reduction in ettringite content is observed with increasing SMS dosage, as the OH^−^ promotes the transformation of ettringite into less stable, more readily decomposed forms [81,82,83]. Moreover, SMS reduces the amount of free water within the concrete, thereby slowing the reaction between C_3_A and gypsum and consequently retarding ettringite formation. When the SMS dosage reaches 9‰ and 12‰, cracks appear in the ITZ and a large number of pores are observed in the matrix, particularly in S12, where pronounced porosity is accompanied by flocculent rather than gel-like C-S-H. The dehydration–condensation of SMS generates additional water, while the silane network membrane hinders water transport, resulting in locally excessive or insufficient water-to-binder ratios and, consequently, pore formation. Together with the reduced ettringite content, these effects ultimately lead to the deterioration of macroscopic performance.

**Figure 16:**
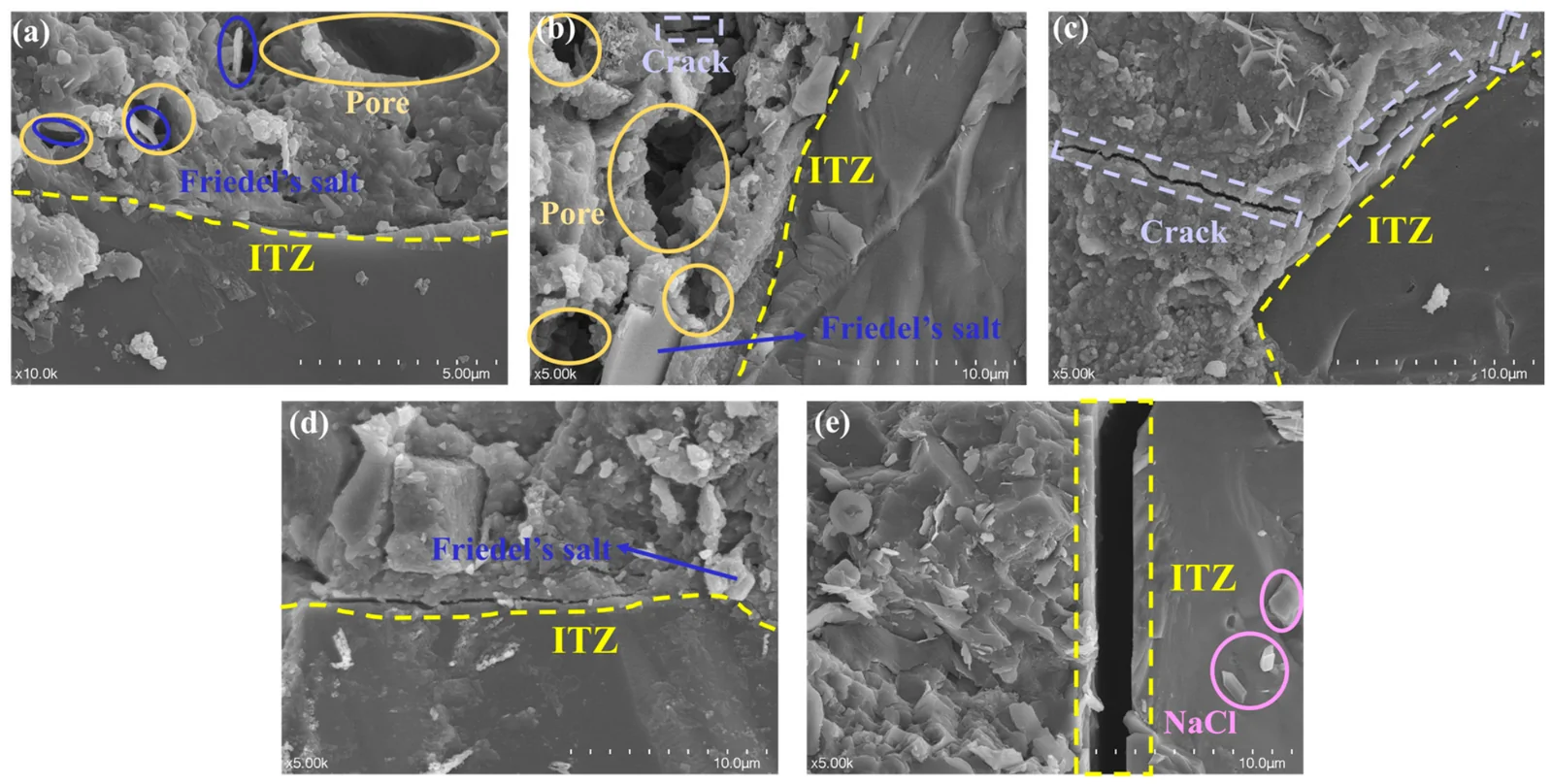
SEM images of ITZs after 30 cycles: (**a**) S0; (**b**) S3; (**c**) S6; (**d**) S9; (**e**) S12.

The micromorphology after 30 dry–wet cycles is shown in Figure 16. The matrix of S0 and S3 experiences significant deterioration, characterized by the formation of numerous pores and cracks. Additionally, flattened hexagonal crystalline forms of Friedel’s salt are distinctly observed, a marked contrast to the microstructure noted before the cycling process. Nevertheless, such degradation is typical for concrete exposed to chloride salt dry–wet cycles. A dense matrix not only provides high strength but also impedes chloride penetration through reduced porosity and enhanced chloride-binding capacity. However, under prolonged cyclic exposure, the expansive calcium chloroaluminate hydrates and Friedel’s salt generate considerable pore pressure. The dense matrix hinders the release of this pressure, resulting in the formation of pores and cracks. In the later stage of cycling, the alkalinity of the matrix decreases, leading to the transformation of ettringite into calcium chloroaluminate hydrates and Friedel’s salt into Kuzel’s salt, thereby aggravating the development of pores and cracks. In contrast, the mortar integrity of S6, S9, and S12 remains significantly higher, accompanied by a reduced formation of corrosion products such as Friedel’s salt. However, NaCl crystallization is observed, and the cracks at the ITZ progressively widen, indicating that SMS alters the degradation mode of RFAC under chloride salt dry–wet cycling. The -CH_3_ groups not only hinder the ingress of the erosive solution but also isolate chloride ions from the mortar matrix, thereby providing effective protection. Meanwhile, they impede water transport during hydration, reducing the compactness of hydration products, which renders the inherently weak ITZ the critical vulnerable zone under cyclic exposure. This not only leads to a decline in mechanical performance but also creates preferential pathways for rapid chloride penetration. By comparison, S9 exhibits the densest microstructure after cycling, with minimal cracking, negligible Friedel’s salt formation, and no NaCl crystallization, indicating the highest resistance to chloride salt degradation.

#### 3.6.2. XRD

**Figure 17:**
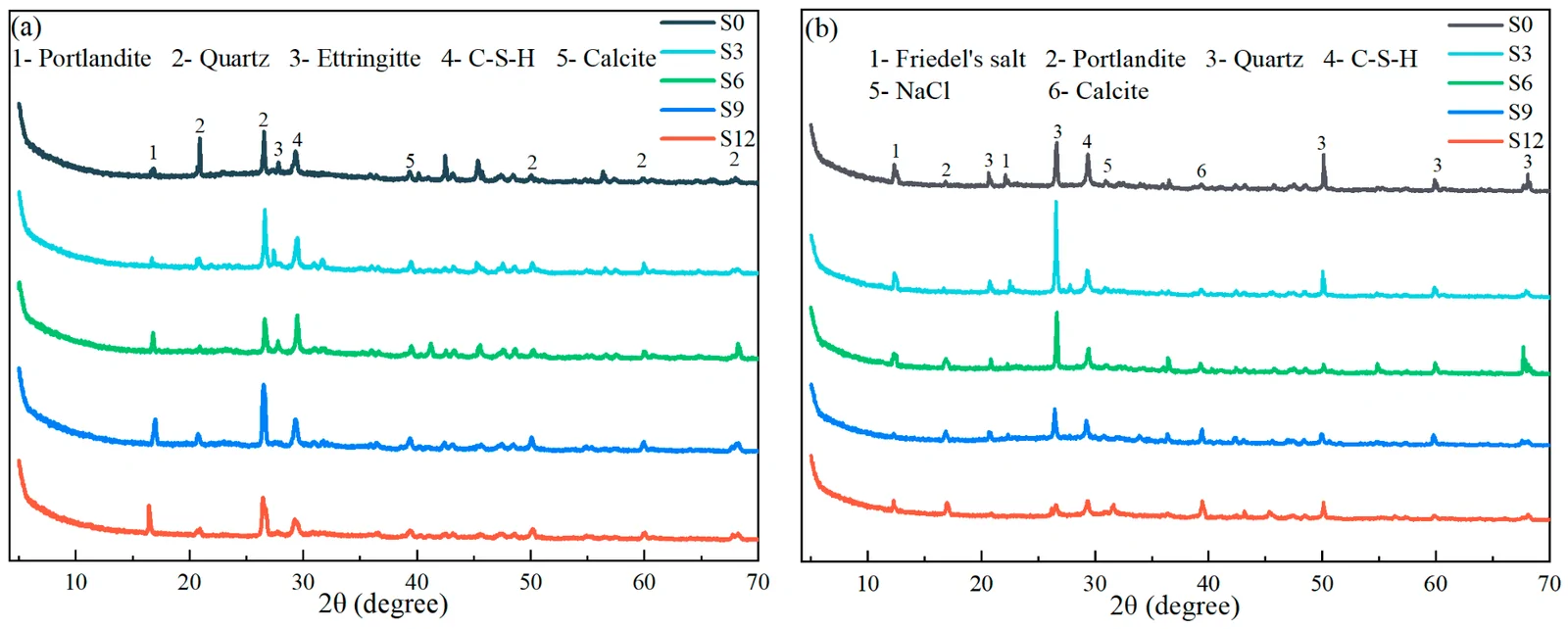
XRD of RFAC before (**a**) dry–wet cycling and after 30 cycles (**b**).

The phase compositions of RFAC with varying SMS dosages before and after chloride salt dry–wet cycles are examined by XRD, as presented in Figure 17. Prior to cycling, the phase assemblages of different specimens are comparable to those of conventional concrete, consisting primarily of portlandite (Ca(OH)_2_), calcite (CaCO_3_), C–S–H, ettringite, and quartz (SiO_2_), although differences in relative peak intensities are observed. The incorporation of SMS increases the diffraction intensity of portlandite. This may be related to the alkaline environment provided by SMS and the reduced consumption of portlandite during secondary hydration caused by limited water availability. At appropriate SMS dosages, S3 and S6 exhibit stronger C–S–H-related diffraction signals, which can be attributed to enhanced secondary hydration of mineral admixtures due to sufficient hydration products and reduced competition for water between recycled fine aggregate and fresh mortar after SMS modification [84]. However, at excessive SMS content, the hydrophobic layer may restrict water migration and limit further hydration. Therefore, the weakened C–S–H peak in S12 indicates that excessive SMS addition inhibits hydration processes [61,85].

After 30 dry–wet cycles, Friedel’s salt formation is observed in all mixtures, while its diffraction intensity gradually decreases with increasing SMS dosage. This trend suggests that SMS reduces the formation of chloride-binding products, which may be associated with reduced chloride ingress caused by the hydrophobic barrier and the silane-derived surface film. However, excessive SMS addition does not continuously improve durability performance. In S12, the enlarged ITZ cracks and increased pore connectivity provide preferential pathways for external aggressive agents, leading to the detection of NaCl diffraction peaks after cycling.

In addition, the decrease in portlandite peaks accompanied by the increase in calcite peaks after cycling suggests carbonation-related phase evolution. This phenomenon is more pronounced in S12, which may be associated with the unfavorable pore structure induced by excessive SMS incorporation. The increased pore connectivity facilitates the transport of CO_2_ and chloride ions, which may contribute to enhanced carbonation-related phase evolution and chloride-induced deterioration. Therefore, an appropriate SMS dosage is required to achieve a balance between hydrophobic protection and microstructural stability. Among all mixtures, S9 exhibits the weakest Friedel’s salt diffraction intensity while maintaining favorable hydration characteristics, demonstrating its superior resistance to chloride salt erosion.

#### 3.6.3. MIP Analysis

**Figure 18:**
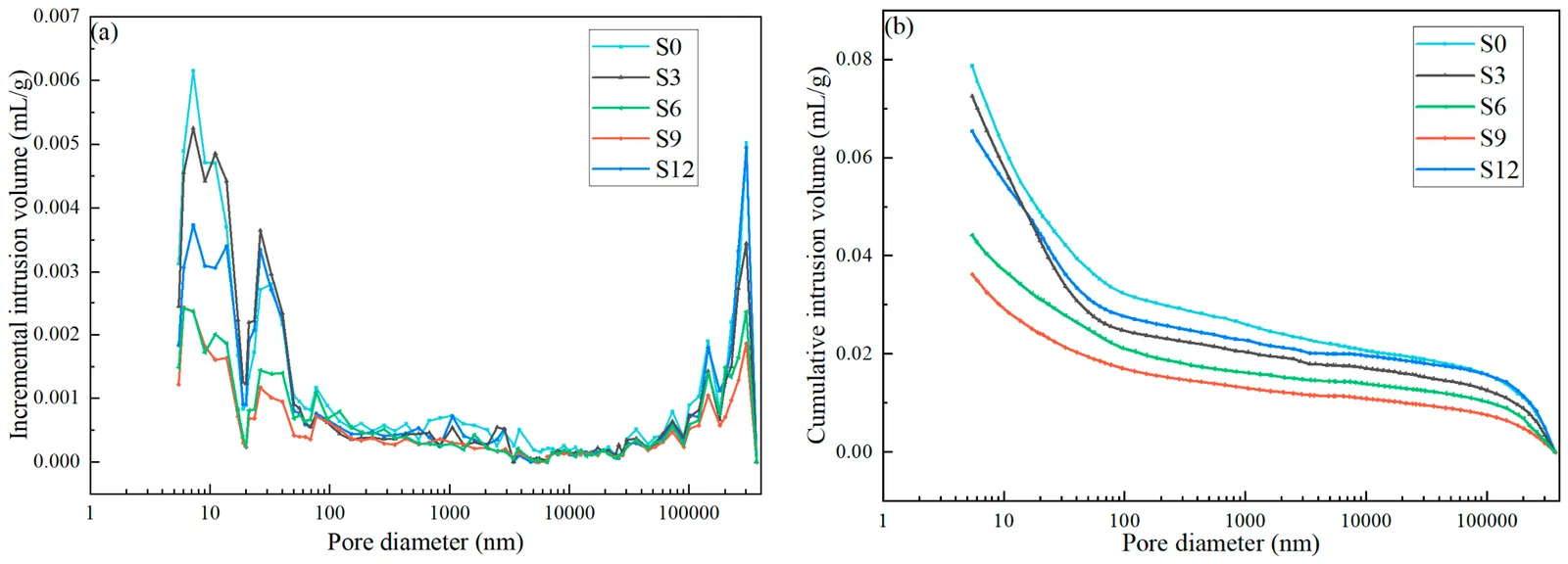
The curves of (**a**) cumulative pore volume and (**b**) cumulative pore volume after 30 dry–wet cycles.

The pore-size distribution and cumulative porosity of specimens after 30 dry–wet cycles are shown in Figure 18. The pore-size distribution exhibits two distinct peaks, corresponding to ultra-micropores and micro-capillary pores (≤200 nm) and non-capillary pores (≥100,000 nm), while pores in the intermediate range of meso-capillary and macro-capillary pores are scarce. Owing to the hydrophobic properties conferred by SMS, RFAC demonstrates suppressed water ingress and transport, which subsequently restricts chloride penetration and ensures the matrix structure remains dense even after the cycling process. This results in a reduction in pore quantity across all pore size ranges, particularly in specimens S6 and S9, which thereby achieve an optimal balance between hydrophobicity and structural compactness.

In contrast, excessive SMS addition (S12) produces an adverse effect. Although enhanced hydrophobicity limits external water penetration, excessive restriction of moisture migration during hydration reduces matrix densification. Consequently, enlarged ITZ cracks and highly connected macropores are generated, providing preferential pathways for chloride ions and other aggressive species. Therefore, improving hydrophobicity alone is insufficient to ensure durability enhancement, and an appropriate SMS dosage is required to maintain pore structure stability. In this study, S9 achieved the most favorable balance between hydrophobic protection and pore structure characteristics.

Moreover, as shown in Figure 18b, SMS effectively affects the total porosity of RFAC, exhibiting an initial decrease followed by an increase with increasing SMS dosage, with the value of S12 approaching that of S0. This evolution reflects the competition between beneficial and adverse effects induced by SMS incorporation. At moderate dosages, SMS provides hydrophobic protection while maintaining favorable hydration and microstructural development, resulting in reduced porosity. However, excessive SMS addition restricts moisture availability and inhibits hydration, leading to increased pore formation and higher porosity.

## 4. Mechanism Analysis

### 4.1. Degradation of the Hydrophobic Membrane

#### 4.1.1. XPS

**Figure 19:**
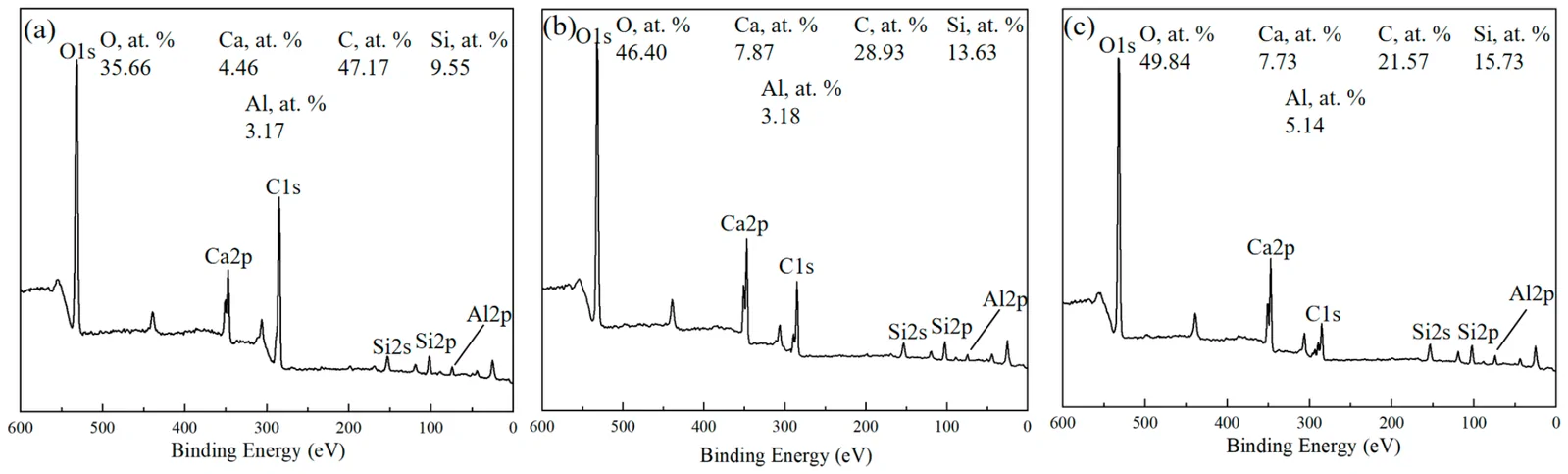
Full-spectrum XPS spectra of different samples before dry–wet cycles: (**a**) S3; (**b**) S9; (**c**) S12.

The elemental and chemical composition of the hydration paste on the RFAC surface is investigated using XPS, as presented in Figure 19. The main elements detected on the RFAC surface are oxygen, silicon, and carbon, accompanied by minor amounts of calcium and aluminum. As the SMS dosage increases, the relative quantity of C1s decreases, while the relative quantities of O1s, Si2s, and Si2p increase. This trend is consistent with the findings of Wang et al. [86], who reported similar behavior during hydrophobic modification using methyl-functionalized compounds. The reduction in the C1s signal does not indicate a decrease in the absolute carbon content of the hydration paste. Rather, it reflects the introduction of additional oxygen and silicon sources by SMS, which resulted in a decreased relative proportion of carbon. These observations suggest that SMS underwent effective dehydration and condensation, followed by covalent grafting onto the outer surfaces of C-S-H, Ca(OH)_2_, and silicate phases, thereby forming a hydrophobic membrane.

**Figure 20:**
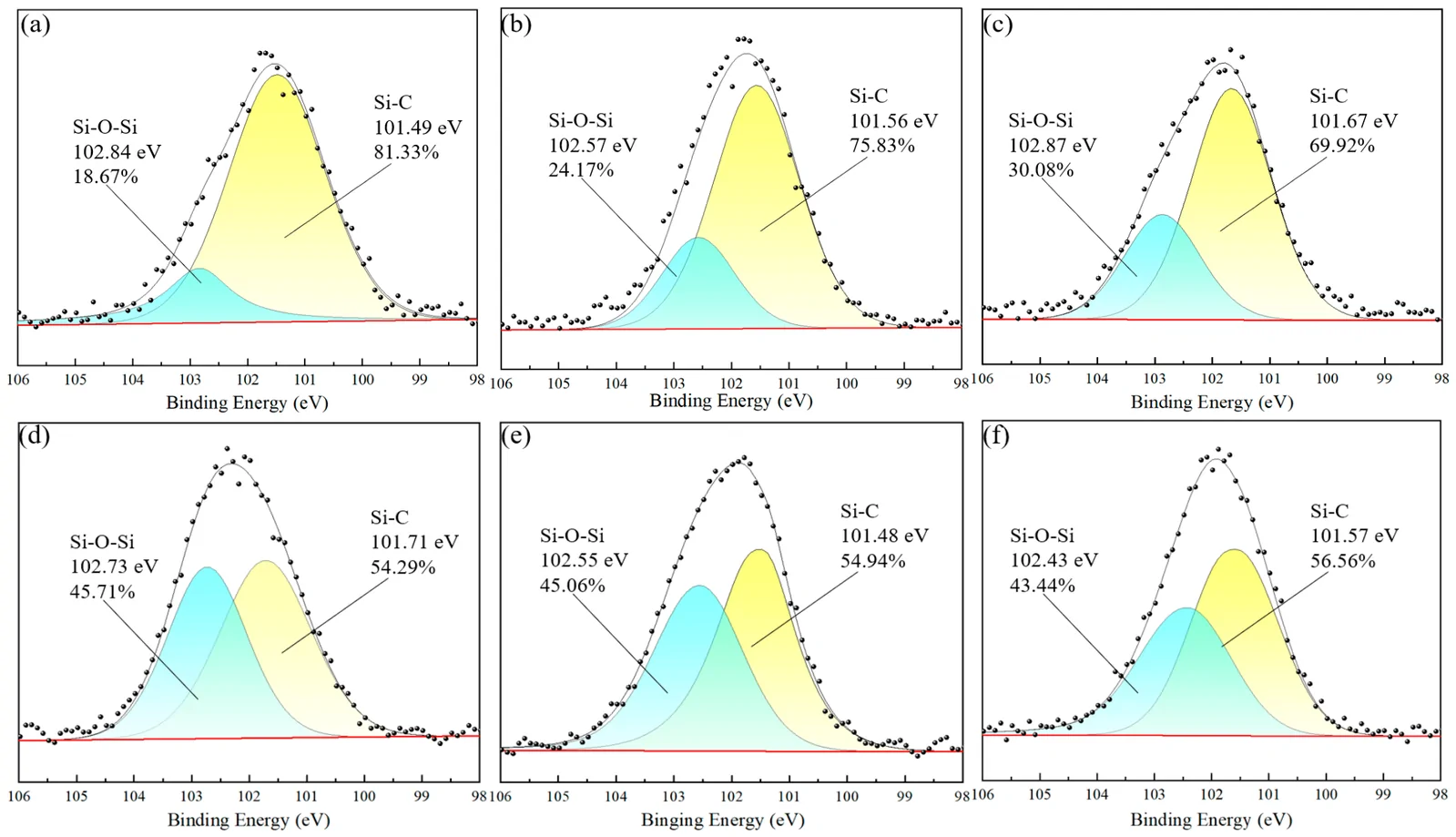
Si2p high-resolution XPS spectra of different samples: (**a**–**c**) S3, S9, S12 before dry–wet cycling and (**d**–**f**) S3, S6, S9 after 30 dry–wet cycles.

As shown in Figure 20, curve-fitting and deconvolution of the Si2p narrow spectra before and after dry–wet cycling are performed to identify subpeaks, and the corresponding binding energies are analyzed to elucidate the degradation mechanism of the hydrophobic film under chloride salt cycling. The binding energies at 101.4 eV and 102.8 eV are assigned to Si-C bonds located on the outer layer and Si-O-Si bonds within the siloxane network, respectively. These findings indicate that SMS underwent effective hydrolysis and polycondensation on the surfaces of hydration products such as C-S-H and Ca(OH)_2_, ultimately forming a silane-based network structure with terminal hydrophobic -CH_3_ groups [87,88,89,90]. With increasing SMS content, the proportion of Si-O-Si bonds in specimens S9 and S12 increases by 5.5% and 5.91%, respectively, due to the formation of a more extensive siloxane backbone [58]. Following 30 dry–wet cycles, the proportion of Si-C bonds decreases by 27.04%, 20.89‰, and 12.42% in S3, S9, and S12, respectively, while the relative content of Si-O-Si increases. This suggests that the outer Si-C bonds within the silane network are cleaved and -CH_3_ groups were detached. The observed degradation can be attributed to the nucleophilic attack of chloride ions on the weakly polar Si-C bonds, particularly during the wetting phase. In addition, the alkaline environment of concrete promotes the transformation of Si-C bonds into Si-O or Si-OH bonds. Furthermore, calcium leaching from C-S-H during the wetting phase may destabilize the Si-O-Si framework, leading to a decrease in bonding energy [91]. The binding energy of Si-O-Si decreases by 0.11 eV, 0.02 eV, and 0.44 eV in S3, S9, and S12, respectively, after 30 cycles, indicating a reduction in interatomic bonding strength and the degree of condensation of the silane network [58]. This structural degradation is also considered a key factor contributing to the decline in the water contact angle of RFAC after cycles.

#### 4.1.2. FT-IR

**Figure 21:**
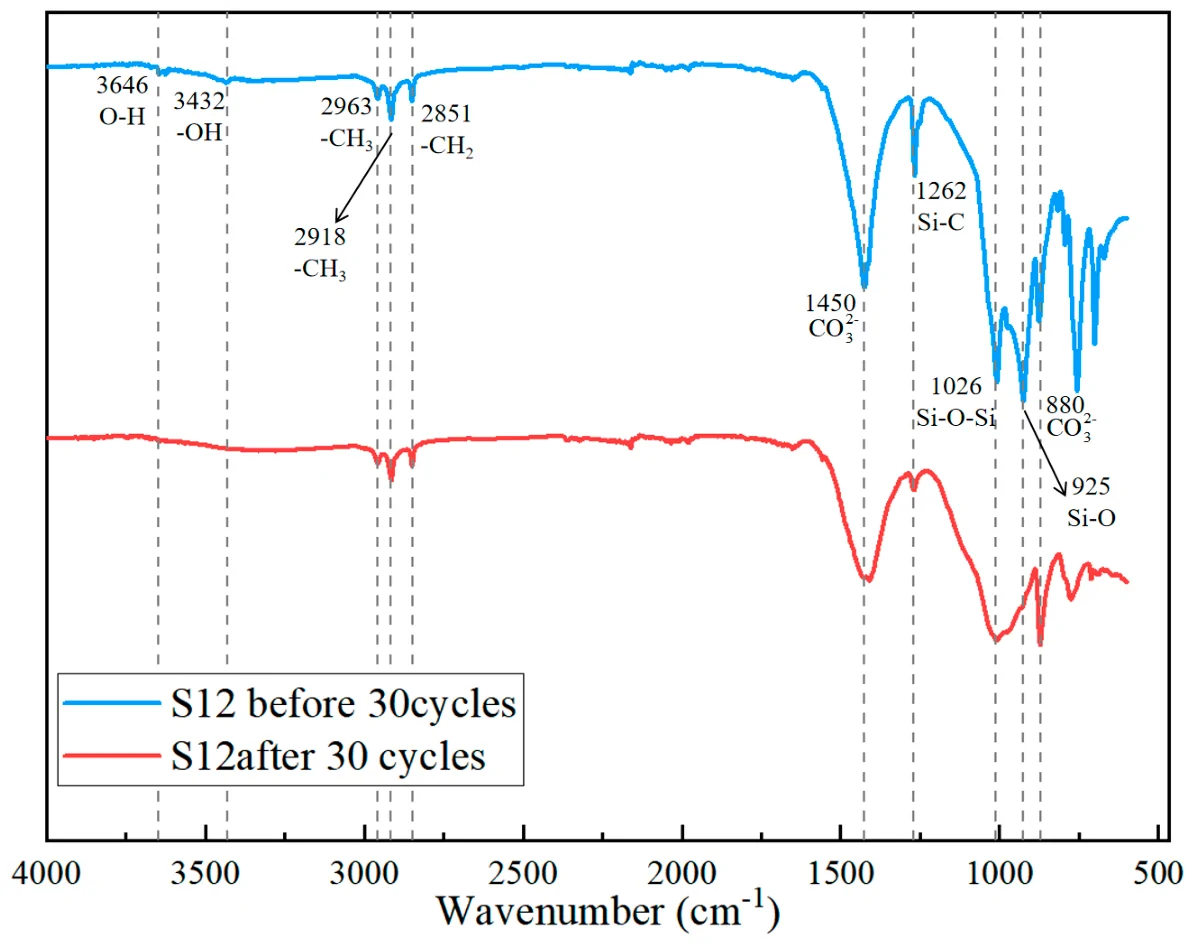
FT-IR of S12 before dry–wet cycling and after 30 cycles.

**Figure 22:**
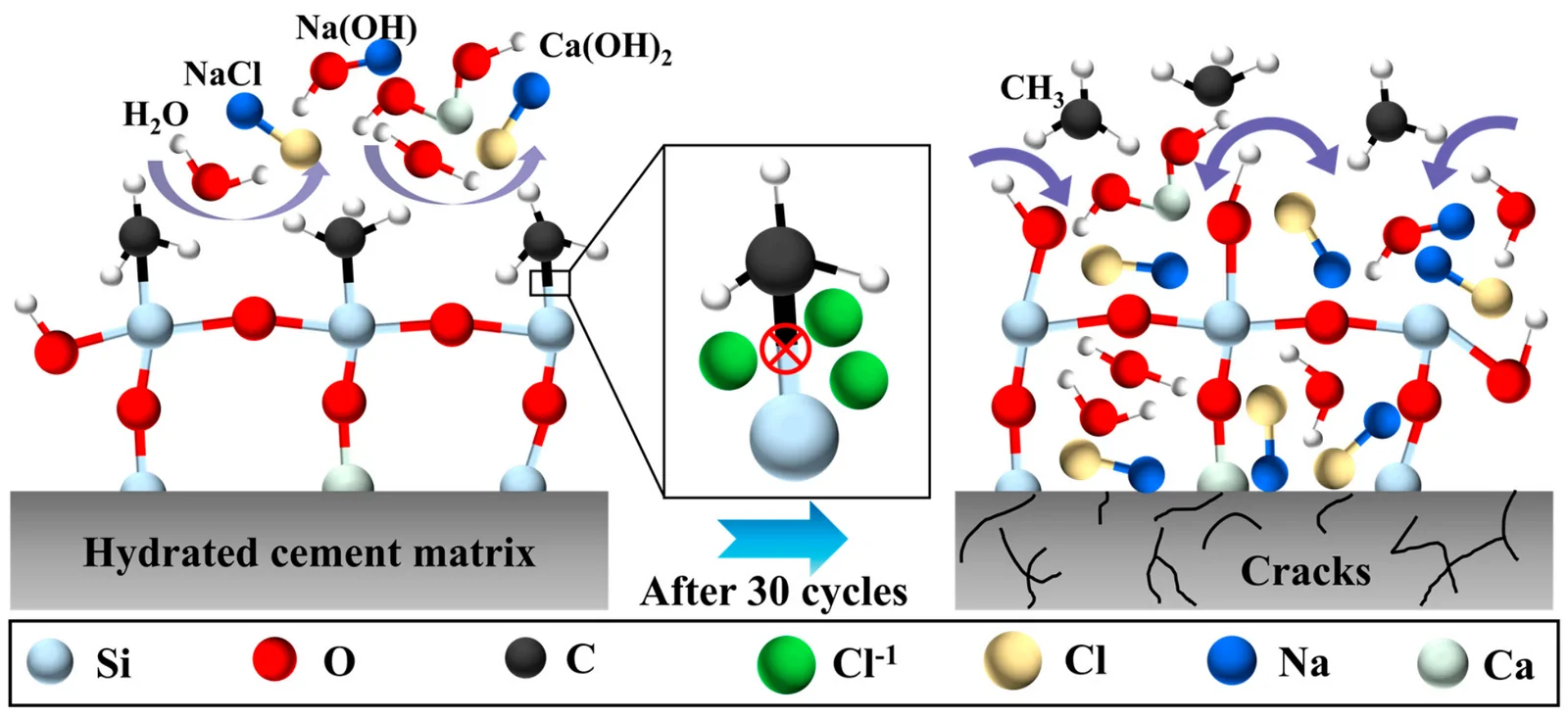
Degradation mechanism of the silane-based network film.

The S12 specimen was selected for FT-IR testing, as shown in Figure 21. Pronounced absorption peaks are observed for S12 at 2963, 2918, and 2851 cm^−1^ both before and after cycling, which are attributed to the symmetric and asymmetric stretching vibrations of –CH_3_ and –CH_2_ groups [92,93,94]. The presence of these characteristic peaks indicates that hydrophobic organic groups remain attached to the matrix surface before and after cycling, confirming the successful hydrophobic modification by SMS [84]. After cycling, the intensities of these absorption peaks decrease slightly, suggesting partial alteration or loss of surface hydrophobic structures. The absorption band at 1026 cm^−1^ is assigned to the asymmetric stretching vibration of Si–O–Si, originating from the siloxane network formed through dehydration–condensation reactions between SMS and cement hydration products, such as C–S–H and Ca(OH)_2_ [31,94]. Due to the highly alkaline environment of concrete, particularly within non-hydrophobic regions, the Si–O–Si network may undergo partial structural degradation or rearrangement, resulting in changes in peak intensity after cycling [95]. The most pronounced variation is observed at the absorption band around 1262 c^−1^, which is associated with Si–C stretching vibrations [31,96]. The substantial decrease in this peak intensity after cycling indicates that the Si–C-related structures are more susceptible to deterioration during chloride salt dry–wet exposure, which is consistent with the XPS results. Considering the relatively lower stability of Si–C linkages compared with the siloxane framework, repeated wetting–drying processes and chloride-containing environments may accelerate the degradation of Si–C-related structures. As the linkage between hydrophobic groups and the siloxane backbone, the weakening of Si–C structures may reduce the stability of terminal hydrophobic moieties, thereby impairing the ability of the siloxane network to maintain effective surface hydrophobicity. Although the characteristic peaks of –CH_3_ and –CH_2_ groups remain detectable after cycling, their presence alone is insufficient to ensure long-term hydrophobic protection once the connection between hydrophobic groups and the inorganic framework is weakened. Consequently, water penetration into the matrix may gradually increase, accelerating chloride-induced deterioration. The proposed degradation process of the SMS-induced hydrophobic network under chloride dry–wet cycling is illustrated in Figure 22.

In summary, the deterioration of the SMS-induced hydrophobic film under chloride exposure is mainly associated with the weakening of Si–C-related structures and the gradual loss of hydrophobic barrier effectiveness. Although the hydrophobic groups and the siloxane framework remain partially preserved after cycling, structural changes at the Si–C-related linkages may reduce the stability of the hydrophobic network and contribute to the deterioration of surface hydrophobicity.

### 4.2. Mechanistic Discussion of SMS Modification and Penetration Characteristics

#### 4.2.1. Mechanistic Discussion of SMS Modification

The water absorption and desorption rates (Figure 10 and Figure 11) indicate that SMS markedly suppresses moisture migration during dry–wet cycling. The incorporation of SMS improves the resistance of RFAC against chloride-induced deterioration, which can be associated with the combined effects of surface hydrophobic modification and favorable microstructural characteristics at an appropriate dosage. The increased contact angle indicates that SMS reduces the surface wettability of RFAC, thereby decreasing water penetration and limiting chloride transport. Meanwhile, the variations in hydration-related phases identified by XRD and FTIR, together with the pore characteristics obtained from MIP analysis, suggest that SMS may influence the evolution of the cementitious matrix. Similar effects have been reported for silicate-based modifiers, which can regulate surface properties and modify pore characteristics in cement-based materials. Therefore, the enhanced durability of SMS-modified RFAC is considered to result from the synergistic effects of reduced moisture transport and improved microstructural stability.

**Figure 23:**
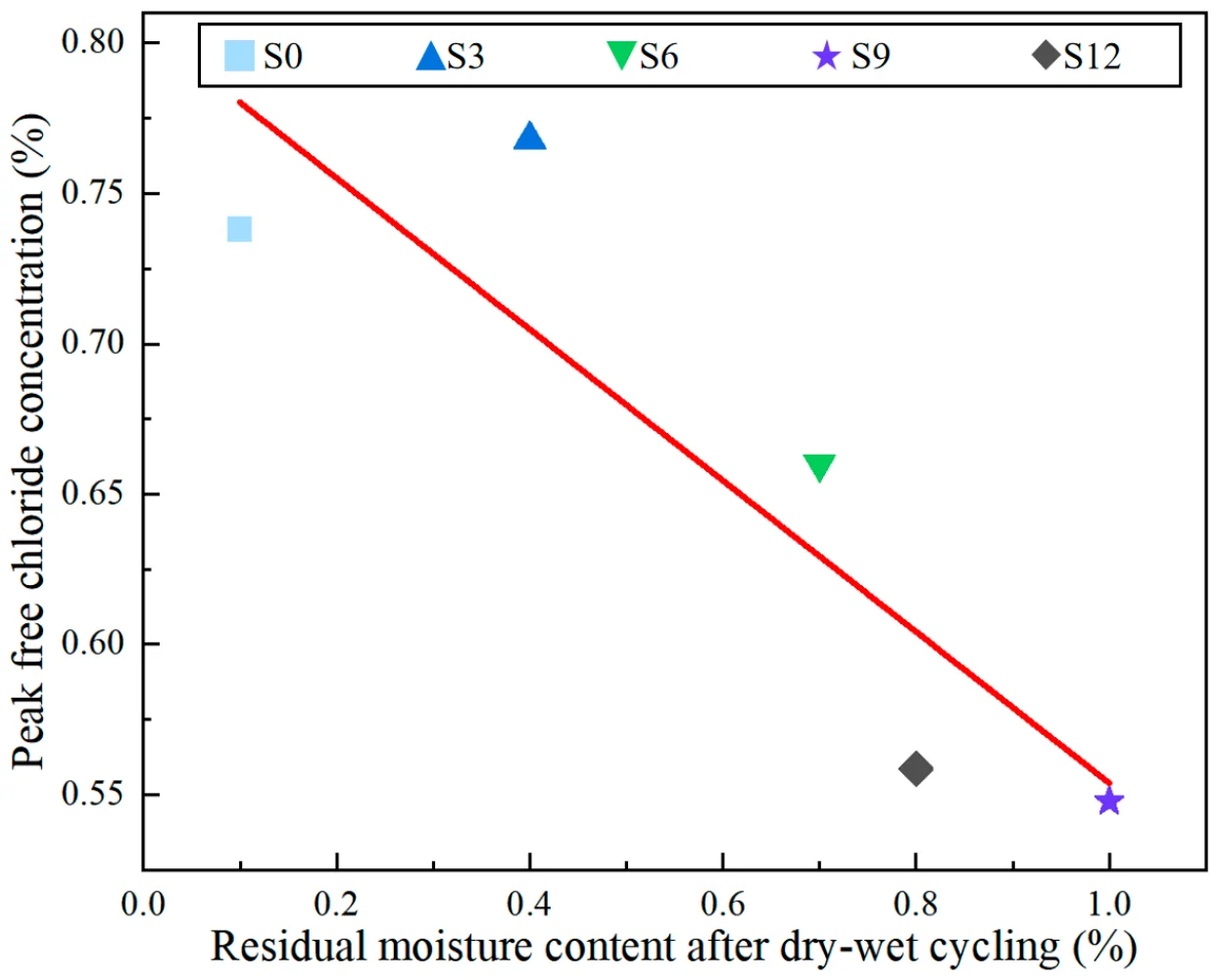
Relationship between residual moisture content and peak free chloride concentration.

Compared with S0, the water absorption rates of S3–S12 at the 30th cycle are reduced by 9.5%, 16.3%, 35.0%, and 27.9%, respectively, while the corresponding desorption rates decrease by 15.9%, 28.9%, 53.8%, and 42.8%, respectively. The residual moisture content of RFAC is defined as the difference between the water absorption rate and desorption rate at the 30th cycle, and its relationship with the peak free chloride concentration is established, as shown in Figure 23. As the residual moisture content increases, the peak free chloride concentration exhibits a decreasing trend, which is consistent with the findings of Chang et al. [24]. These results suggest that SMS modifies the moisture retention behavior of RFAC during wetting–drying cycles. A lower moisture retention variation provides a weaker moisture gradient during the wetting stage, thereby reducing capillary-driven absorption and limiting chloride solution penetration depth [24,76]. During drying, water preferentially evaporates while chloride ions remain within the pore solution, resulting in chloride accumulation at specific depths and the formation of concentration peaks characteristic of convection-dominated transport. With increasing internal relative humidity, the moisture gradient between the pore solution and external environment decreases, suppressing evaporation and weakening capillary-driven chloride migration. Meanwhile, higher internal relative humidity reduces chloride concentration gradients in the pore solution and suppresses chloride accumulation induced by repeated drying processes.

Moreover, the silane-based network film isolates chloride ions from the hydration matrix, resulting in fewer binding products, such as Friedel’s salt, thereby mitigating enhanced diffusion associated with the accumulation of bound chlorides. In contrast, excessive hydration inhibition in S12 leads to pronounced cracking at the interfacial transition zone, providing preferential pathways for free chloride transport, and should therefore be avoided.

#### 4.2.2. Penetration Characteristics

The above experimental results demonstrate that the incorporation of SMS increases the contact angle of the RFAC matrix, thereby reducing the water absorption, dehydration rate, and capillary rise, which in turn decreases the free chloride ion content and penetration depth. In fact, concrete is a typical multi-porous material, and chloride ions penetrate into the interior of RFAC along with water through micro-capillary channels. Based on the theory of capillary suction, the capillary absorption height was calculated using Equation (2).

(2) $$h=\frac{2\sigma \cos\theta }{r_{0}\rho g}$$

where *h* denotes the capillary absorption height (mm), *σ* represents the surface tension of water (7.80 × 10^−2^ N/m), *ρ* is the density of saturated NaCl solution (1200 kg/m^3^), *g* denotes the gravitational acceleration (9.8 N/kg), *θ* represents the contact angle between water and the mortar (°), and *r*_0_ is the average radius of the capillary pores (nm).

Capillary pores with diameters of 10–200 nm are considered [97,98], and the average capillary pore diameter of different specimens is calculated from the MIP results using Equation (3):

(3) $$r_{0}=\frac{\sum_{i}(r_{i}\cdot \Delta V_{i})}{\sum_{i}\Delta V_{i}}$$

where *r_i_* denotes the representative pore diameter of the pore size interval, and Δ*V_i_* represents the incremental mercury intrusion volume corresponding to that interval.

The capillary absorption height calculated using Equation (2) is presented in Table 4.

The calculated results appear counterintuitive. Although S6 and S9 exhibit considerable resistance to chloride salt ingress, their capillary absorption behavior results in relatively high capillary rise heights, which are 46.4% and 41.2% higher than that of S0, respectively. In addition, the calculated values for all specimens exceed the experimental results, indicating that the liquid fails to reach static equilibrium. Under such conditions, the direct application of Equation (2) is inappropriate, as the contact angle employed corresponds to the post-erosion surface wettability, whereas the hydrophobic RFAC effectively inhibits chloride penetration, allowing the internal matrix to retain favorable hydrophobicity. Therefore, the Lucas–Washburn equation is adopted, and the contact angle is further modified to account for the capillary absorption behavior of hydrophobic RFAC under chloride salt dry–wet cycling.

By comparing the contact angle, XPS, and FT-IR results of the specimens before and after cycling, it is evident that chloride salt dry–wet cycling induces the cleavage of Si-C bonds. Accordingly, an erosion factor describing the degradation of Si-C bonds as a function of free chloride ion content is introduced, as expressed in Equation (4):

(4) $$\eta \left(x\right)=\frac{C_{f}\left(x\right)-C_{f,\min}}{C_{f,\max}-C_{f,\min}}$$

where *C_f_*(*x*) (%wt.conc) denotes the free chloride ion content at a depth of *x* (mm), and *C_f_*_,min_ and *C_f_*_,max_ represent the minimum and maximum values of the free chloride ion content, respectively.

In this model, the pore structure of RFAC is simplified as an equivalent capillary channel system, and the heterogeneous pore network is represented by an equivalent capillary radius. This assumption allows the complex pore characteristics of recycled aggregate concrete to be described using a simplified theoretical framework. Meanwhile, the influence of SMS modification is considered through the hydrophobic correction factor, which reflects the reduction in water affinity caused by the surface modification.

Accordingly, a contact angle distribution function is formulated, as given in Equation (5):

(5) $$\cos\theta \left(x\right)=\cos\theta_{i}-\eta \left(x\right)\left(\cos\theta_{i}-\cos\theta_{s}\right)$$

where *θ_s_* is the surface contact angle and *θ_i_* is the internal contact angle, which is taken as the contact angle measured prior to cycling.

By taking the spatial average along the capillary channel, the effective contact angle (*θ_eff_*) within the capillary rise height H is defined as follows:

(6) $$\cos\theta_{eff}=\frac{1}{H}\int_{0}^{H}\cos\theta \left(x\right)d_{x}$$

The classical capillary absorption theory, namely the Lucas–Washburn equation, is expressed as Equation (7):

(7) $$h^{2}\left(t\right)=\frac{\sigma r_{0}\cos\theta_{eff}}{2\mu \tau }t$$

where μ denotes the dynamic viscosity of the liquid, which is taken as 1.15 × 10^−3^ Pa/s by considering the saturated NaCl solution and the experimental temperature, and τ is the resistance correction factor, which is inversely determined from the capillary absorption height H of the reference specimen S0 and is taken as 156.

The calculated values of *cosθ_ef_* and *h* are summarized in Table 5. The parameter *D* is defined as the difference between the experimentally measured capillary absorption height *H* (Figure 14a) and the calculated height *h*, and it is used to evaluate the connectivity of the pore structure within the matrix.

The modified contact angle combined with the Lucas–Washburn equation provides a reasonable description of the penetration behavior of chloride-containing solution in hydrophobic RFAC under chloride salt dry–wet cycling. Negative D values (S6 and S9) indicate that the experimental capillary absorption heights are lower than the calculated values, suggesting that liquid transport is restricted compared with ideal capillary absorption. This deviation may be associated with the combined effects of SMS-induced hydrophobicity, increased pore tortuosity, ink-bottle-type pores, and partially disconnected pore structures, which limit the effective penetration of liquid into the matrix. This behavior reflects the balance between the hydrophobic protection provided by SMS and its influence on hydration and pore development. In contrast, S12 exhibits a relatively large positive D value, indicating that excessive SMS incorporation weakens the agreement between theoretical and experimental penetration behavior. Although S12 possesses high hydrophobicity, excessive inhibition of hydration may result in increased pore connectivity, more continuous cracks, and a less compact matrix, thereby providing preferential pathways for aggressive solution transport. The study by Tittarelli et al. [99] demonstrated that the water-repellent characteristics of hydrophobic concrete may facilitate gas transport through dry pores, thereby increasing oxygen availability and potentially affecting reinforcement corrosion behavior. Similarly, S12 exhibits reduced water absorption but increased pore connectivity, indicating that excessive hydrophobic modification may not necessarily provide optimal corrosion protection under chloride exposure. Therefore, the application of internal hydrophobic agents should consider not only the improvement of water repellency but also their influence on pore structure and connectivity. The quantitative relationship established between contact angle and free chloride ion content represents a preliminary evaluation of the influence of hydrophobic modification on chloride transport. Further investigations considering reinforcement corrosion behavior and long-term field exposure are still required.

It should be noted that the proposed model was established based on the capillary absorption behavior of the chloride-containing solution in the SMS-modified recycled fine aggregate concrete investigated in this study. The model parameters may vary with changes in aggregate type, pore structure characteristics, cementitious composition, and surface modification methods. Therefore, further validation is required before extending this model to other cement-based materials. Nevertheless, the proposed model provides a theoretical approach for evaluating the influence of hydrophobic modification on moisture and chloride transport behavior. The findings are generally consistent with previous studies on hydrophobic modification of cement-based materials, where durability improvement was mainly attributed to reduced water transport and favorable modification of pore characteristics. However, compared with ordinary concrete systems reported previously, recycled fine aggregate concrete contains more porous recycled particles and weaker interfacial transition zones. Therefore, the improvement achieved by SMS in this study is attributed to the combined effects of hydrophobic modification and improved resistance of the recycled aggregate concrete matrix against moisture and chloride transport.

## 5. Conclusions

In this study, sodium methyl silicate was employed as a hydrophobic agent for RFAC, and the degradation behavior of the SMS-induced hydrophobic network during chloride salt dry–wet cycling, the mechanism of durability enhancement, and the penetration characteristics of chloride-containing solutions in RFAC were investigated. The following conclusions are drawn:

1. Sodium methyl silicate improves the durability of RFAC under chloride salt dry–wet cycling, with an optimal dosage of 9‰. After 30 cycles, the compressive strength and RDEM increase by 12.9‰ and 17.6%, respectively, while the peak free chloride ion content, chloride erosion depth, and convection zone depth decrease by 25.8%, 52.7%, and 80.0%, respectively. In addition, RFAC with 9‰ sodium methyl silicate exhibits a relatively dense microstructure with lower porosity and reduced chloride-related products.
2. With increasing sodium methyl silicate dosage, the mechanical properties of RFAC initially increase and then decrease, whereas hydrophobicity shows a continuous increase with diminishing improvements. Excessive SMS incorporation (12‰) may inhibit hydration and adversely affect the interfacial transition zone, resulting in increased pore connectivity, reduced strength, and accelerated chloride transport. Therefore, an appropriate SMS dosage is required to balance hydrophobic protection and microstructural stability.
3. Chloride salt dry–wet cycling gradually reduces the contact angle of SMS-modified RFAC. The deterioration of the SMS-induced hydrophobic network is mainly associated with the weakening of Si–C-related structures, while the Si–O–Si framework remains relatively stable. These structural changes contribute to the gradual reduction in surface hydrophobicity during long-term cycling.
4. Sodium methyl silicate modifies moisture transport behavior within RFAC by reducing both water ingress and moisture exchange during dry–wet cycling. This effect maintains a relatively stable internal humidity condition, reduces water uptake during wetting, and suppresses chloride accumulation and salt crystallization, thereby improving resistance against chloride-induced deterioration.
5. A quantitative relationship between the contact angle of hydrophobic RFAC and the free chloride ion content is established, and the modified Lucas–Washburn model reasonably describes the capillary absorption behavior of SMS-modified RFAC under the investigated conditions.

From an engineering perspective, the optimal SMS dosage identified in this study provides a practical reference for improving the durability of recycled fine aggregate concrete exposed to water and chloride environments, such as marine structures and chloride-exposed underground concrete facilities. However, the optimal dosage may vary depending on aggregate characteristics, cementitious composition, pore structure, and environmental conditions. Therefore, further field investigations and long-term exposure tests are required to evaluate the applicability of the proposed dosage under different service conditions.

## Figures and Tables

**Table 1:** Chemical compositions and physical properties of cementitious material.

Components	Cement	FA	SF	GGBS
Chemical composition (%)				
SiO_2_	22.86	52.51	86.20	34.50
Al_2_O_3_	5.44	31.14	1.08	17.70
Fe_2_O_3_	4.37	3.66	–	1.64
CaO	61.91	3.82	0.22	34.00
MgO	1.03	1.13	0.79	1.03
SO_3_	1.84	1.75	0.83	–
TiO_2_	0.26	0.97	–	–
K_2_O	0.81	1.69	–	–
MnO	0.12	0.04	0.02	0.36
Physical properties				
Specific surface area (m^2^/kg)	365	360	23,000	429
Apparent density (kg/m^3^)	3130	2500	2350	3100

Note: “–“ is not a measured item.

**Table 2:** Mix proportions of concrete (kg/m^3^).

Specimen	NCA	RFA	Cement	Water	SP	FA	SF	GGBS	SMS
S0	1119	534	354	168	3	76	25	51	-
S3	1119	534	354	168	3	76	25	51	1.52
S6	1119	534	354	168	3	76	25	51	3.04
S9	1119	534	354	168	3	76	25	51	4.55
S12	1119	534	354	168	3	76	25	51	6.07

**Table 3:** The difference between capillary absorption height and chloride ion penetration depth.

Number of Cycles	ΔH (mm) = Capillary Absorption Height − Chloride Ion Penetration Depth				
	S0	S3	S6	S9	S12
5	1.1	0.9	0.7	0.4	0.3
10	1.4	1.1	0.9	0.7	0.8
15	0.8	0.9	0.8	0.9	1.2
20	0.6	0.3	0.8	0.4	0.5
25	0.5	0.2	0.6	0.1	−1
30	0.3	0.2	0.2	0.1	−1.3

**Table 4:** Height of calculated capillary water absorption.

Samples	*r*_0_ (nm)	*θ* (°)	*h* (mm)
S0	30	27.8	389.1
S3	26	39.2	392.9
S6	17	43	569.6
S9	14	54.3	549.6
S12	20	54.9	381.4

**Table 5:** Comparison between calculated and experimental values.

Samples	*cosθ_eff_*	*h* (mm)	*H* (mm)	*D* (mm)
S0	–	16.0	16.0	0
S3	42.3	13.3	13.2	0.1
S6	45.6	10.4	10.3	−0.1
S9	62.2	7.7	7.3	−0.4
S12	63.2	9.1	10.4	1.3

## Data Availability

The original contributions presented in this study are included in the article. Further inquiries can be directed to the corresponding author.
